# Preferences on the Use of Prokinetic Agents in Adult Intensive Care Unit Patients—An International Survey

**DOI:** 10.1111/aas.70045

**Published:** 2025-04-24

**Authors:** Vera Crone, Morten Hylander Møller, Waleed Alhazzani, Lasse Grønningsæter, Abdulrahman Al‐Fares, Johanna Hästbacka, Marlies Ostermann, Carmen A. Pfortmueller, Ricard Ferrer, Annika Reintam Blaser, Martin I. Sigurdsson, Olof Wall, Eric Keus, Wojciech Szczeklik, Paul J. Young, Chris McGrath, Maurizio Cecconi, Anders Perner, Mette Krag

**Affiliations:** ^1^ Department of Intensive Care Holbæk Hospital Holbæk Denmark; ^2^ Department of Intensive Care Copenhagen University Hospital ‐ Rigshospitalet Copenhagen Denmark; ^3^ Department of Clinical Medicine University of Copenhagen Copenhagen Denmark; ^4^ Health Research Centre Ministry of Defence Health Services Riyadh Saudi Arabia; ^5^ Critical Care and Internal Medicine Department, College of Medicine Imam Abdulrahman Bin Faisal University Dammam Saudi Arabia; ^6^ Department of Anesthesiology, Division of Emergencies and Critical Care Oslo University Hospital Oslo Norway; ^7^ Department of Anaesthesia, Critical Care Medicine and Pain Medicine Al‐Amiri Hospital, Ministry of Health Kuwait City Kuwait; ^8^ Department of Intensive Care Tampere University Hospital, Wellbeing Services County of Pirkanmaa and Tampere University Tampere Finland; ^9^ Department of Critical Care, King's College London Guys and St. Thomas Hospital London UK; ^10^ Department of Intensive Care Inselspital, Bern University Hospital and University of Bern Bern Switzerland; ^11^ Vall D'Hebron University Hospital. SODIR Research Group, VHIR, Medicine Department Barcelona Autonomous University Barcelona Spain; ^12^ Department of Anaesthesiology and Intensive Care University of Tartu Tartu Estonia; ^13^ Department of Intensive Care Medicine Lucerne Cantonal Hospital Lucerne Switzerland; ^14^ Department of Anaesthesiology and Intensive Care Medicine Landspital ‐ The National University Hospital of Iceland Reykjavik Iceland; ^15^ Faculty of Medicine University of Iceland Reykjavik Iceland; ^16^ Department of Anaesthesiology and Intensive Care Danderyds Sjukhus Stockholm Sweden; ^17^ Department of Critical Care University Medical Center Groningen Groningen the Netherlands; ^18^ Center for Intensive Care and Perioperative Medicine Jagiellonian University Medical College Krakow Poland; ^19^ Intensive Care Unit Wellington Hospital Wellington New Zealand; ^20^ Medical Research Institute of New Zealand Wellington New Zealand; ^21^ Department of Critical Care Belfast Health and Social Care Trust Belfast UK; ^22^ Biomedical Sciences Department Humanitas University Milan Italy; ^23^ Department of Anaesthesia and Intensive Care IRCCS‐Humanitas Research Hospital Rozzano Italy; ^24^ Department of Anaesthesia, Centre of Head and Orthopaedics Copenhagen University Hospital Copenhagen Denmark

**Keywords:** feeding intolerance, intensive care, prokinetic agents, survey

## Abstract

**Introduction:**

Feeding intolerance complicates enteral nutrition in intensive care unit (ICU) patients but is poorly defined. Prokinetic agents are administered to facilitate the uptake of enteral nutrition, but preferences for their use among clinicians in ICUs are unknown.

**Methods:**

We conducted an international electronic survey targeting ICU doctors. The survey included 76 questions that focused on symptoms considered when assessing feeding intolerance, preferences for using prokinetic agents, and willingness to participate in a future randomised trial on prokinetic agents.

**Results:**

We received 830 responses from 17 countries, with an overall response rate of 29%. Most respondents were specialists working in mixed ICUs. Feeding intolerance was assessed by 90% of respondents in their clinical work, though only 36% considered it well defined. Gastric residual volume and vomiting were symptoms most frequently used for defining feeding intolerance. Metoclopramide was the preferred prokinetic agent (54% of respondents), followed by erythromycin (42%). Four out of five considered using combination therapy, primarily a combination of metoclopramide and erythromycin (89%). Concerns about side effects were reported for all agents, with extrapyramidal symptoms and QT prolongation being the most common across agents. The majority (91%) of respondents supported a future randomised trial comparing prokinetic agents to placebo.

**Conclusion:**

This international survey found practice variations in the symptoms reportedly used to assess feeding intolerance. Metoclopramide was the preferred prokinetic agent, followed by erythromycin. Most respondents supported a future randomised trial.

## 1 Introduction

Feeding intolerance in critically ill patients is associated with worse clinical outcomes [1, 2]. Despite this, feeding intolerance remains poorly defined and includes a broad range of symptoms [3]. As a result, there is no standardised method for monitoring feeding intolerance, leading to reported prevalences in the literature ranging from 2% to 75% [3].

Prokinetic agents enhance upper gastrointestinal motility, facilitating the movement and absorption of nutrients [4] and they are administered to improve the delivery of enteral nutrition in patients with feeding intolerance. However, studies addressing their use vary in indication, agent type, dosage, and frequency [5].

With this survey, we aimed to investigate the preferences for the use of prokinetic agents among doctors in ICUs internationally.

We hypothesised variations in the preferred clinical practice of administering prokinetic agents and in the symptoms considered when assessing feeding intolerance.

## 2 Methods

### 2.1 Study Design, Data Collection and Approvals

We conducted a cross‐sectional online survey among doctors in ICUs internationally. We used the secure web application Research Electronic Data Capture (REDCap) [6] hosted by the Region of Zealand, Denmark. The survey was tested and revised by four researchers and six ICU doctors at three different sites before data collection. We distributed the survey on the 1 September 2024 and closed it by the 13 October 2024. Participation was voluntary, and respondents did not receive any financial compensation for the participation. Completion of the survey was considered informed consent. Respondents could register their work email. This was used to calculate response rates for specific sites and avoid duplicate responses. The survey was approved by the Legal Department of Scientific Research in the Region of Zealand (approval number: REG‐036‐2024). Other approvals were waived in Denmark because no patient data were collected, and the work emails collected from the doctors were optional. Where applicable, national investigators obtained the necessary approvals before distributing the survey.

We prepared this manuscript in accordance with the Consensus‐Based Checklist for Reporting of Survey Studies (CROSS) checklist (Appendix S1) [7].

### 2.2 Survey Description

The survey consisted of 76 questions, including 22 main questions. The first five questions addressed details on department and hospital characteristics as well as the level of education of respondents. The following five main questions focused on the symptoms clinicians evaluated in their daily assessment of feeding tolerance. This was followed by nine main questions addressing the use of prokinetic agents. Lastly, one question focused on the use of guidelines, and two main questions evaluated the potential for a future trial. Branching logic was applied based on prior responses where relevant. This ensured that only respondents who answered ‘yes’ to a specific question were presented with the subsequent questions. Consequently, some questions were answered by only a subset of participants. The survey was designed with a target time of 10 min to complete. The questions were dichotomous (yes/no), predefined multiple choice, and one free‐text question. The survey was conducted in English with no translations. The complete distributed survey is available in Appendix S2.

### 2.3 Survey Distribution

The survey aimed to reach a broad range of ICU doctors to ensure a sample size large enough to report trends in the responses. This was attempted through distribution in an established international research network, the Collaboration for Research in Intensive Care (CRIC) [8]. International doctors were invited to serve as national investigators and coordinated the distribution of the survey link to their national networks (Appendix S3). We distributed two reminders before the database was closed. Each national investigator reported the total number of doctors who received the survey link in their respective countries.

### 2.4 Statistical Analysis

We present all results descriptively. Categorical variables are presented as numbers with corresponding percentages and 95% confidence intervals (CIs). Continuous variables are reported as medians with interquartile ranges (IQRs). Missing data for each main question are reported, and no imputation of missing data was made. We included all responses in our analysis. All analyses and graphical designs were made in R version 4.4.2 and Microsoft Excel.

## 3 Results

We received 830 responses from 17 different countries. Most respondents were specialist doctors working in mixed ICUs within public hospitals (Table 1).

**TABLE 1:** Characteristics of respondents.

	*N*	Percentages (95% CI)
**Country** ^a^		
Denmark	289	34.9% (31.7%–38.3%)
Spain	76	9.2% (7.3%–11.3%)
Norway	61	7.4% (5.7%–9.4%)
Sweden	57	6.9% (5.3%–8.8%)
Finland	55	6.6% (5%–8.6%)
Saudi Arabia	53	6.4% (4.8%–8.3%)
Great Britain	38	4.6% (3.3%–6.3%)
Poland	37	4.5% (3.2%–6.1%)
Estonia	35	4.2% (3%–5.9%)
Switzerland	33	4.0% (2.8%–5.6%)
Northern Ireland	26	3.1% (2%–4.6%)
Kuwait	18	2.2% (1.3%–3.4%)
Italy	16	1.9% (1.1%–3.1%)
Iceland	12	1.5% (0.8%–2.5%)
New Zealand	11	1.3% (0.7%–2.4%)
The Netherlands	9	1.1% (0.5%–2.1%)
Canada^b^	1	0.1% (0.0%–0.7%)
Other^c^	1	0.1% (0.0%–0.7%)
**Clinical position**		
Specialist	745	90.0% (87.8%–91.9%)
**Type of hospital**		
Public general	459	55.3% (51.8%–58.7%)
Public specialist	354	42.7% (39.3%–46.1%)
Private general hospital	9	1.1% (0.5%–2.1%)
Private specialist hospital	8	1.0% (0.4%–1.9%)
**Type of ICU**		
Mixed ICU	694	83.7% (81.0%–86.2%)
Cardiothoracic ICU	32	3.9% (2.7%–5.4%)
Medical ICU	38	4.6% (3.3%–6.2%)
Neurological/neurosurgical ICU	43	5.2% (3.8%–6.9%)
Surgical ICU	22	2.7% (1.7%–4.0%)
Number of beds pr ICU^d^	8	(6–15)

*Note:* Number of respondents in total = 830. Counts with percentages and 95% confidence interval or median with interquartile range.

^a^ Two respondents had not reported country.

^b^ One reported to be based in Canada, though survey was not distributed in Canada.

^c^ One respondent reported other without further specification.

^d^ Fourteen respondents had not stated number of beds in their ICU.

The overall response rate was 29%, with country‐specific response rates ranging from 8% to 100% (Appendix S4).

Overall, missing data for the main question was below 2%. The Appendix S5 includes detailed information on missingness.

### 3.1 Feeding Intolerance and Symptoms Assessed

Of 828 respondents, 294 (36%, 95% CI: 32%–39%) considered feeding intolerance well‐defined, while 535 (65%, 95% CI: 61%–68%) did not. Most respondents (*n* = 747, 90%, 95% CI: 88%–92%) indicated that they assessed feeding intolerance in their daily clinical practice, and 95% (95% CI: 93%–96%) considered feeding intolerance a clinically relevant outcome when evaluating the effect of prokinetic agents.

**FIGURE 1:**
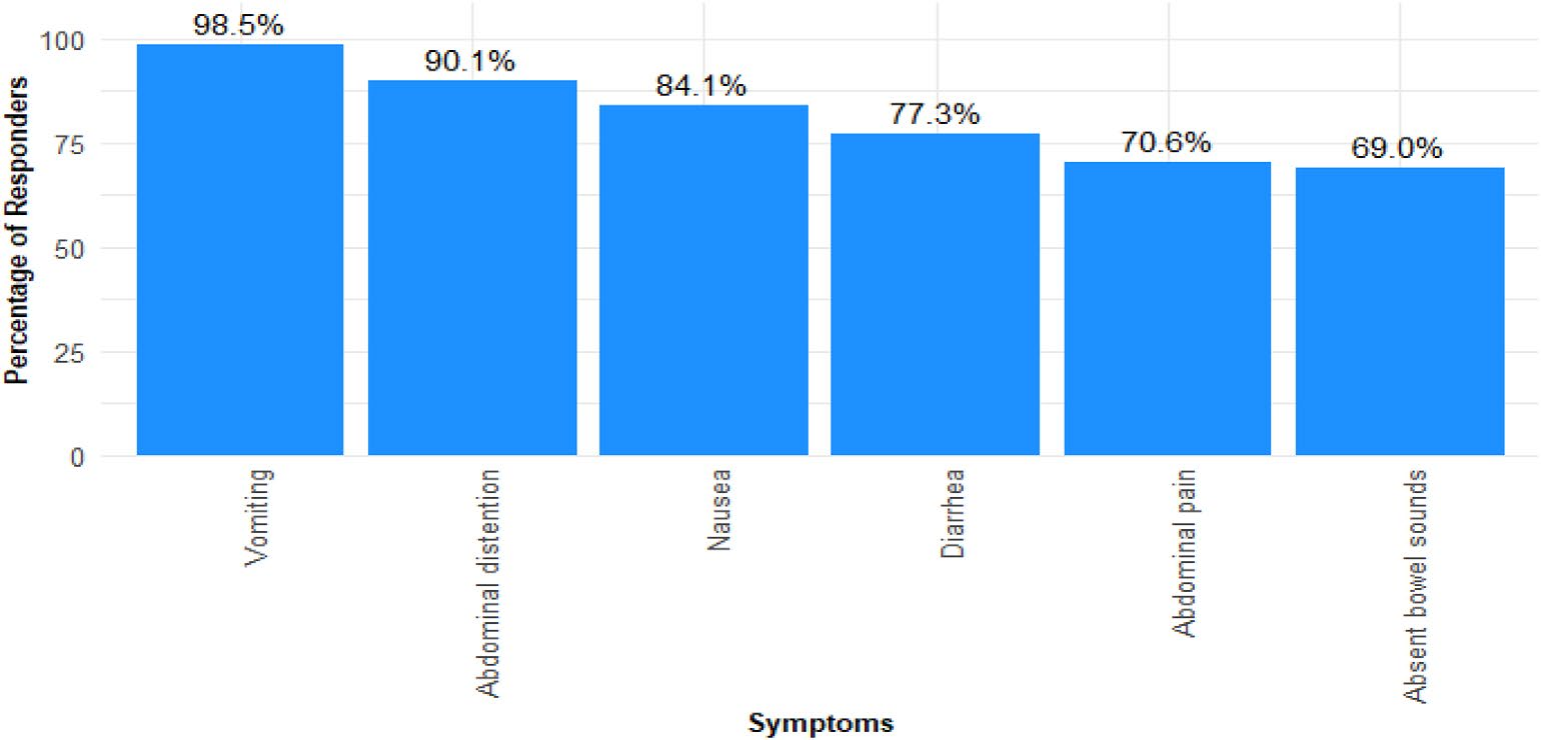
Gastrointestinal symptoms considered when assessing feeding intolerance. It was possible to select more than one symptom. Some chose to respond only to certain symptoms, leaving other symptoms unaddressed (numbers and percentages in Supporting Information). Missingness was less than 2%.

Figure 1 and Appendix S6 illustrate the symptoms evaluated in the assessment. Of 725 respondents, 318 (44%, 95% CI: 40%–48%) considered all six symptoms when assessing feeding intolerance, and 95% (95% CI: 93%–96%) considered at least three of the symptoms.

Of the respondents assessing feeding intolerance, the majority (89%, 95% CI: 87%–91%) reported using gastric residual volume as a diagnostic measure, with a volume of > 500 mL being the most reported threshold (68%) followed by > 200 mL (25%) (Appendix S7).

Around half of the respondents (*n* = 397, 54%, 95% CI: 50%–57%) reported insufficient feeding as a symptom of feeding intolerance. For more than half of these respondents (*n* = 218), the threshold for feeding intolerance was receiving less than 50% of the planned daily enteral nutrition.

### 3.2 Guidelines

Approximately half of the respondents (51%, 95% CI: 47%–54%) reported following a guideline when prescribing prokinetic agents, with the majority using a local guideline (Appendix S8).

### 3.3 Prokinetic Agents

Most clinicians (93%, 95% CI: 91%–95%) indicated using prokinetic agents to treat feeding intolerance in their clinical practice. Two‐thirds of clinicians used prokinetic agents therapeutically, while one‐third reported using them both therapeutically and prophylactically. The time to initiation of treatment ranged from zero to 13 days of symptoms, with a median of 2 days (IQR 1–2).

Metoclopramide was the first‐choice prokinetic agent for 54% of respondents (95% CI: 50%–57%), and erythromycin was the preferred second choice for 48% (95% CI: 44%–52%). Both agents were predominantly administered intravenously (85% and 95%, respectively). Data on the first choice prokinetic agent by country are shown in Appendix S9.

Approximately 5% of respondents preferred alternative prokinetic agents, with neostigmine and laxatives being the most frequently mentioned (Appendix S10).

Neostigmine was reported to be used as a prokinetic agent, albeit not for bowel obstruction, by 34% of respondents (95% CI: 31%–38%).

**FIGURE 2:**
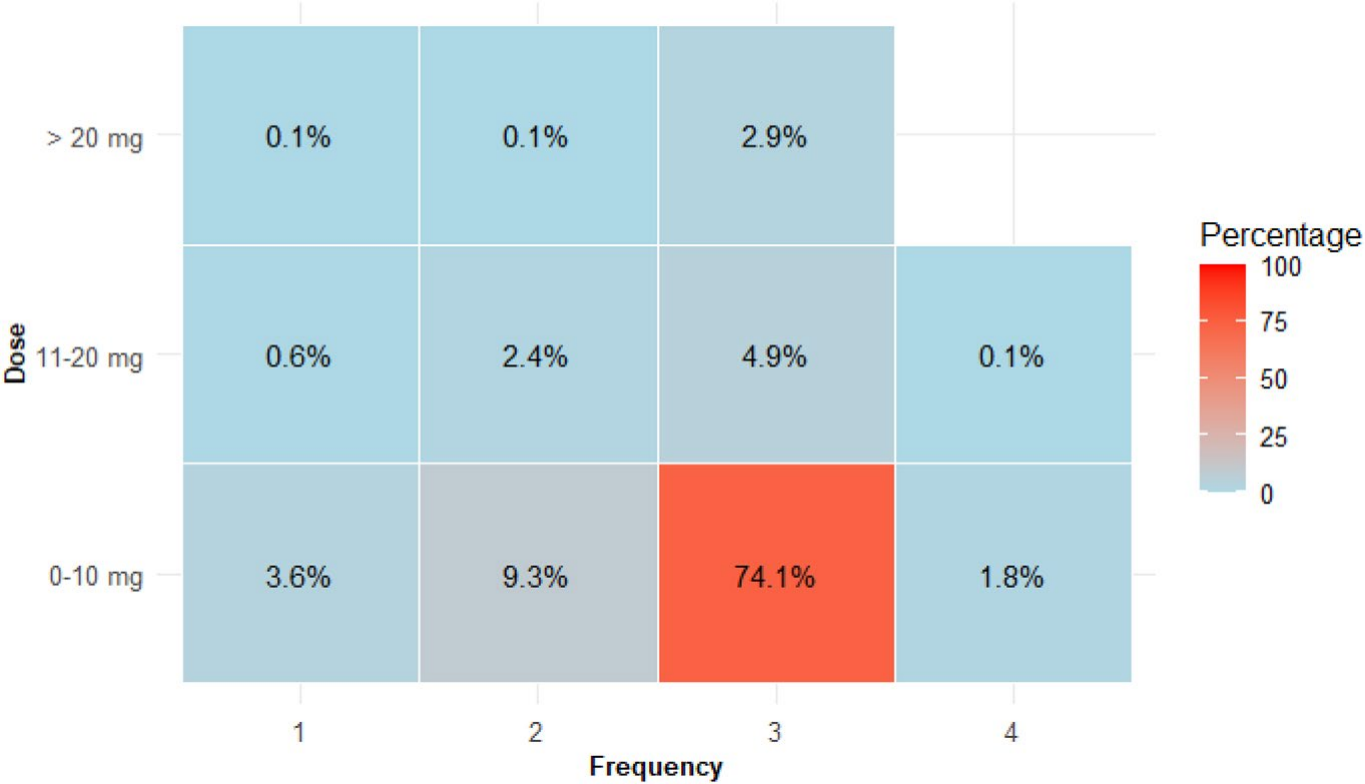
The reported doses and frequencies of metoclopramide selected as the preferred first or second choice. Total number of respondents = 726.

**FIGURE 3:**
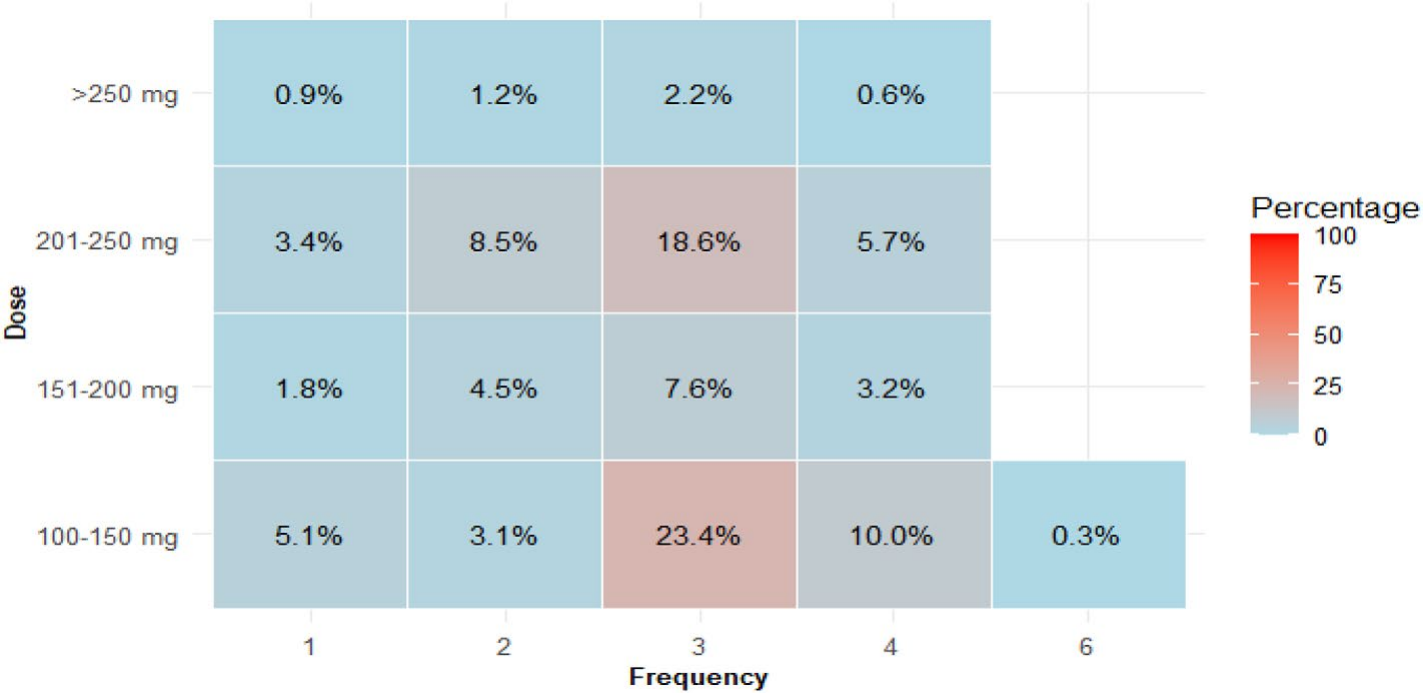
The reported doses and frequencies of erythromycin selected as preferred first or second choice. Total number of respondents = 692.

The preferred (74%, 95% CI: 71%–77%) regimen of metoclopramide was up to 10 mg three times daily for 3 days (Figure 2 and Appendix S11). In contrast, the preferred dose of erythromycin was variable, as 23% (95% CI: 20%–27%) preferred 100–150 mg and 19% (95% CI: 16%–22%) preferred 250 mg, both three times daily for 3 days (Figure 3 and Appendix S11). Data on domperidone and prucalopride are available in Appendix S12.

### 3.4 Side Effects

**FIGURE 4:**
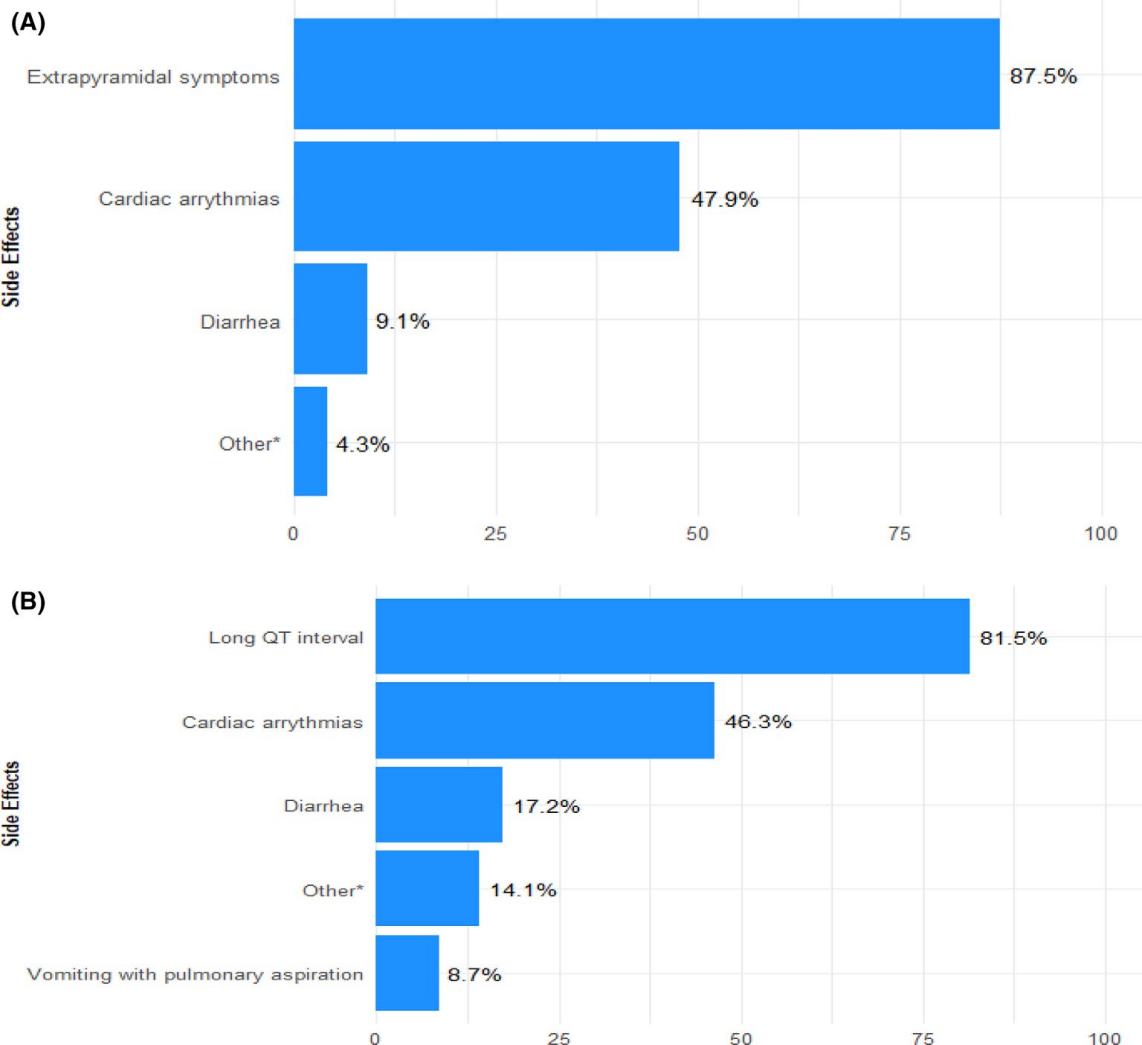
(A) Reported concerns about side effects when prescribing metoclopramide. Total number of respondents = 582. It was possible to select more than one side effect. * The main concerns under the category of ‘other’ were cerebral symptoms, abdominal pain and delirium. (B) Reported concerns about side effects when prescribing erythromycin. Total number of respondents = 460. It was possible to select more than one side effect. *The main concerns mentioned under the ‘other’ category were drug interactions, antibiotic resistance, abdominal pain and allergy.

When prescribing prokinetic agents, 80% (95% CI: 77%–83%) reported concerns about side effects associated with metoclopramide, with extrapyramidal symptoms being the predominant concern, followed by cardiac arrhythmias (Figure 4a). For erythromycin, 67% (95% CI: 63%–70%) of respondents expressed concerns about side effects, primarily prolonged QT, followed by cardiac arrhythmias (Figure 4b).

### 3.5 Combination Therapy

Four out of five clinicians considered combination therapy, with the majority (89%, 95% CI: 87%–92%) indicating a preference for combining metoclopramide and erythromycin. The primary reason for starting combination therapy was the insufficient effect of a single prokinetic agent (78%, 95% CI: 74%–81%).

Twenty‐five percent considered increasing the dose of the prokinetic agent if the initial effect was insufficient.

### 3.6 Future Trial

Ninety‐one percent of respondents expressed willingness to randomise patients in a future trial, with both erythromycin, metoclopramide, and a combination of the two considered relevant interventions (Appendix S13).

## 4 Discussion

In this international survey, we found that most doctors reported feeding intolerance to be poorly defined, with considerable variability in the symptoms assessed. We found that metoclopramide was the preferred prokinetic agent, followed by erythromycin. Most respondents supported a future randomised trial comparing erythromycin and metoclopramide to placebo.

The results of this survey indicate variation in how feeding intolerance is assessed. This was also the case in a previous survey of nurses in ICUs and medical wards, where gastric residual volume and gastrointestinal symptoms were the most commonly used parameters for monitoring feeding intolerance [9]. The lack of a standardised definition may contribute to this variability, as supported by a 2014 systematic review of 72 studies identifying 43 different definitions of feeding intolerance [3].

Our survey revealed that almost 90% of doctors used gastric residual volume as an indicator when assessing feeding intolerance in their daily practice. This aligns with findings from a previous survey of ICUs in the United Kingdom, where 98% of units reported measuring gastric residual volume, with the majority using it to define feeding intolerance [10]. However, a Cochrane review of 1585 adult ICU patients found low certainty of evidence regarding the impact of gastric residual volume monitoring on patient‐relevant outcomes such as mortality, pneumonia, and length of hospital stay [11]. One explanation may be that gastric aspiration is an imprecise measurement method and serves only as a surrogate marker for feeding intolerance [12]. This complexity is also reflected in discrepancies in guidelines on enteral nutrition, which differ in their recommendations for measuring gastric residual volume. The American Society of Critical Care Medicine and the American Society for Parenteral and Enteral Nutrition (ASPEN) recommend against routine gastric residual volume monitoring in ICU patients receiving enteral nutrition due to lack of correlation with the incidence of pneumonia and aspiration [13]. In contrast, the guideline from the European Society for Clinical Nutrition and Metabolism (ESPEN) suggests delaying enteral feeding when gastric residual volume exceeds 500 mL over 6 h and recommends considering prokinetic agents in such cases [14]. The high percentage of doctors using gastric residual volume in our survey may reflect the predominance of European respondents, aligning with ESPEN guidelines [14].

In our survey, metoclopramide was the preferred prokinetic agent, followed by erythromycin. This contrasts with the ESPEN guidelines, where erythromycin is the recommended first‐line therapy in critically ill patients with feeding intolerance (grade B evidence) [14].

Most respondents considered using a combination of erythromycin and metoclopramide if one agent was insufficient. A randomised trial, including 61 patients, found improved success of feeding with combination therapy without an increase in the proportion of major adverse events. However, patients treated with combination therapy had a significantly higher incidence of watery diarrhoea [15]. Based on that study, the ASPEN guidelines do not recommend combination therapy until further research is conducted [13].

Our survey highlighted ICU doctors' concerns about adverse effects associated with prokinetic agents, especially cardiac side effects and extrapyramidal symptoms. However, most knowledge of cardiac side effects arises from studies assessing higher doses and longer durations than those typically used when treating feeding intolerance [16, 17]. Furthermore, a 2016 systematic review on the safety and efficacy of prokinetic agents in critically ill patients, including 13 randomised trials (*n* = 1341), found no explicit reports of cardiac arrhythmias in the included trials [18].

Similarly, studies on neurological side effects related to metoclopramide have primarily been conducted in non‐ICU populations. Data on the occurrence of extrapyramidal symptoms in acutely ill patients are sparse, potentially explained by the challenges of recognising the symptoms in sedated patients [17, 19, 20]. Overall, few randomised trials on prokinetic agents have assessed side effects sufficiently, and a lot of uncertainty exists [5, 17].

The willingness to participate in a potential future randomised trial was high. The respondents preferred to assess the combination of metoclopramide and erythromycin against placebo, followed closely by an assessment of the agents separately versus placebo. These findings support a future randomised trial on the use of prokinetic agents in ICU patients.

### 4.1 Strengths and Limitations

The strengths of this survey include its international format, providing reasonable external validity, and the high proportion of ICU specialists (90%) among respondents. Missingness was below 2% for the main questions.

The survey also has some limitations. First, the overall response rate (29%), though typical for surveys of this type [21, 22], is low and may limit generalisability, as clinicians with a specific interest in the topic are more likely to respond, potentially skewing results. Additionally, in some countries, the survey was distributed to clinicians working in both ICU and anaesthesia, which may have influenced the responses.

Second, the proportion of respondents per country varied, as distribution was managed by national investigators, introducing potential selection bias. Third, the survey design carries an inherent risk of response bias, as responses may reflect what respondents perceive as correct rather than actual clinical practice. Fourth, only 9.8% of respondents were from outside Europe, limiting external validity in non‐European settings.

## 5 Conclusions

This international survey of ICU doctors revealed variations in symptoms considered for assessing feeding intolerance. Metoclopramide was the preferred prokinetic agent, followed by erythromycin, with variability in dose and frequency. Most respondents expressed support for a future randomised trial.

## Author Contributions

Conceptualisation and study design: VC, MHM, WA, ARB, AP and MK; Writing first draft: VC, MHM and MK; Critical review and approval of manuscript: all authors.

## Conflicts of Interest

The Department of Intensive Care at Rigshospitalet‐Copenhagen University Hospital (MHM) has received funding from the Novo Nordisk Foundation and Sygeforsikringen ‘Denmark’ outside the submitted work. A.R.B. has received speaker or consultancy fees from Nutricia and VIPUN Medical and is holding a grant from the Estonian Research Council (PRG1255) outside the submitted work. The Department of Intensive Care, University Hospital, Inselspital, reports grants from Orion Pharma, Abbott Nutrition International, B. Braun Medical AG, CSEM AG, Edwards Lifesciences Services GmbH, Kenta Biotech Ltd., Maquet Critical Care AB, Omnicare Clinical Research AG, Nestle, Pierre Fabre Pharma AG, Pfizer, Bard Medica S.A., Abbott AG, Anandic Medical Systems, Pan Gas AG Healthcare, Bracco, Hamilton Medical AG, Fresenius Kabi, Getinge Group Maquet AG, Dräger AG, Teleflex Medical GmbH, GlaxoSmithKline, Merck Sharp and Dohme AG, Eli Lilly and Company, Baxter, Astellas, AstraZeneca, CSL Behring, Novartis, Covidien, Phagenesis, Cytel and Nycomed outside the project. The money was paid into departmental funds; no personal financial gain applied. Finally, this research was conducted during the tenure of a Health Research Council of New Zealand Clinical Practioner Research Fellowship held by Paul Young. The Medical Research Institute of New Zealand is supported by Independent Research Organisation Funding from the Health Research Council of New Zealand.

## Supporting information


**Data S1** Supporting Information.

## Data Availability

The data that support the findings of this study are available from the corresponding author upon reasonable request.

## References

[1] J. Li , L. Wang , H. Zhang , et al., “Different Definitions of Feeding Intolerance and Their Associations With Outcomes of Critically Ill Adults Receiving Enteral Nutrition: A Systematic Review and Meta‐Analysis,” Journal of Intensive Care 11 (2023): 29. 37408020 10.1186/s40560-023-00674-3 PMC10320932

[2] A. Reintam Blaser , A. M. Deane , J. C. Preiser , Y. M. Arabi , and S. M. Jakob , “Enteral Feeding Intolerance: Updates in Definitions and Pathophysiology,” Nutrition in Clinical Practice 36, no. 1 (2021): 40–49, 10.1002/ncp.10599. 33242218

[3] A. R. Blaser , J. Starkopf , Ü. Kirsimägi , and A. M. Deane , “Definition, Prevalence, and Outcome of Feeding Intolerance in Intensive Care: A Systematic Review and Meta‐Analysis,” Acta Anaesthesiologica Scandinavica 58 (2014): 914–922. 24611520 10.1111/aas.12302

[4] G. Karamanolis and J. Tack , “Promotility Medications ‐ Now and in the Future,” Digestive Diseases 24 (2006): 297–307. 16849857 10.1159/000092883

[5] V. Crone , M. H. Møller , E. S. Bækgaard , et al., “Use of Prokinetic Agents in Hospitalised Adult Patients: A Scoping Review,” Acta Anaesthesiologica Scandinavica 67 (2023): 588–598, 10.1111/aas.14222. 36847067

[6] REDCap, https://redcap.regionsjaelland.dk/redcap_v14.5.17/DataExport/index.php?pid=1082&report_id=ALL&stats_charts=1.

[7] A. Sharma , N. T. Minh Duc , T. Luu Lam Thang , et al., “A Consensus‐Based Checklist for Reporting of Survey Studies (CROSS),” Journal of General Internal Medicine 36 (2021): 3179–3187. 33886027 10.1007/s11606-021-06737-1 PMC8481359

[8] About CRIC – CRIC – Collaboration for Research in Intensive Care, https://www.cric.nu/about‐cric/.

[9] N. A. Metheny , A. C. Mills , and B. J. Stewart , “Monitoring for Intolerance to Gastric Tube Feedings: A National Survey,” American Journal of Critical Care 21 (2012): e33–e40. 22381994 10.4037/ajcc2012647

[10] B. Jenkins , P. C. Calder , and L. V. Marino , “Gastric Residual Volume Monitoring Practices in UK Intensive Care Units: A Web‐Based Survey,” Journal of the Intensive Care Society 25 (2023): 156–163. 38737302 10.1177/17511437231210483 PMC11086716

[11] H. Yasuda , N. Kondo , R. Yamamoto , et al., “Monitoring of Gastric Residual Volume During Enteral Nutrition,” Cochrane Database of Systematic Reviews 9, no. 9 (2021): CD013335. 34596901 10.1002/14651858.CD013335.pub2 PMC8498989

[12] S. J. Diamond , V. Medici , T. W. Rice , and K. Miller , “Should We Stop Using Gastric Residual Volumes?,” Current Nutrition Reports 4 (2015): 236–241.

[13] S. A. McClave , B. E. Taylor , R. G. Martindale , et al., “Guidelines for the Provision and Assessment of Nutrition Support Therapy in the Adult Critically Ill Patient,” Journal of Parenteral and Enteral Nutrition 40 (2016): 159–211. 26773077 10.1177/0148607115621863

[14] P. Singer , A. R. Blaser , M. M. Berger , et al., “ESPEN Practical and Partially Revised Guideline: Clinical Nutrition in the Intensive Care Unit,” Clinical Nutrition 42 (2023): 1671–1689. 37517372 10.1016/j.clnu.2023.07.011

[15] N. Q. Nguyen , M. Chapman , R. J. Fraser , L. K. Bryant , C. Burgstad , and R. H. Holloway , “Prokinetic Therapy for Feed Intolerance in Critical Illness: One Drug or Two?,” Critical Care Medicine 35 (2007): 2561–2567. 17828038 10.1097/01.CCM.0000286397.04815.B1

[16] R. Fiets , J. M. Bos , A. R. T. Donders , et al., “QTc Prolongation During Erythromycin Used as Prokinetic Agent in ICU Patients,” European Journal of Hospital Pharmacy 25 (2018): 118–122. 31157004 10.1136/ejhpharm-2016-001077 PMC6452395

[17] N. Q. Nguyen and S. L. C. Yi Mei , “Current Issues on Safety of Prokinetics in Critically Ill Patients With Feed Intolerance,” Therapeutic Advances in Drug Safety 2 (2011): 197–204. 25083212 10.1177/2042098611415567 PMC4110811

[18] K. Lewis , Z. Alqahtani , L. Mcintyre , et al., “The Efficacy and Safety of Prokinetic Agents in Critically Ill Patients Receiving Enteral Nutrition: A Systematic Review and Meta‐Analysis of Randomized Trials,” Critical Care 20 (2016): 259. 27527069 10.1186/s13054-016-1441-z PMC4986344

[19] A. S. Rao and M. Camilleri , “Review Article: Metoclopramide and Tardive Dyskinesia,” Alimentary Pharmacology & Therapeutics 31 (2010): 11–19. 19886950 10.1111/j.1365-2036.2009.04189.x

[20] N. Q. Nguyen , “Pharmacological Therapy of Feed Intolerance in the Critically Ills,” World Journal of Gastrointestinal Pharmacology and Therapeutics 5 (2014): 148–155. 25133043 10.4292/wjgpt.v5.i3.148 PMC4133440

[21] P. Sivapalan , K. L. Ellekjaer , A. Perner , et al., “Preferences for Albumin Use in Adult Intensive Care Unit Patients With Shock: An International Survey,” Acta Anaesthesiologica Scandinavica 68 (2024): 1234–1243. 39302760 10.1111/aas.14479

[22] K. L. Ellekjaer , P. Sivapalan , S. N. Myatra , et al., “Preferences and Attitudes on Acetate‐ Versus Lactate‐Buffered Crystalloid Solutions for Intravenous Fluid Therapy—An International Survey,” Acta Anaesthesiologica Scandinavica 69 (2025): e14553. 39627945 10.1111/aas.14553

