# Supplementary material for: Preferences on the Use of Prokinetic Agents in Adult Intensive Care Unit Patients—An International Survey
Source: Acta Anaesthesiol Scand. 2025 Apr 24;69(6):e70045. doi: 10.1111/aas.70045 (PMC12022387; doi:10.1111/aas.70045)
Supplement: Supplementary file 1 — Data S1 Supporting Information. [file AAS-69-0-s001.docx]

Preferences on the use of prokinetic agents in adult intensive care unit patients – an international survey

**Authors:** Vera Crone, Morten Hylander Møller, Waleed Alhazzani, Lasse Grønningsæter, Abdulrahman Al-Fares, Johanna Hästbacka, Marlies Ostermann, Carmen A Pfortmueller, Ricard Ferrer, Annika Reintam Blaser, Martin I. Sigurdsson, Olof Wall, Eric Keus, Wojciech Szczekli, Paul J. Young, Chris McGrath, Maurizio Cecconi, Ander Perner, Mette Krag

**Supplementary** **Appendix**

Table of contents

[Supplement 1: Checklist for Reporting Of Survey Studies (CROSS) 2](#_Toc193971501)

[Supplement 2: The distributed survey 5](#_Toc193971502)

[Supplement 3: Participating countries, sites and investigators 15](#_Toc193971503)

[Supplement 4: Data on response rate 17](#_Toc193971504)

[Supplement 5: Missingness in the main questions 18](#_Toc193971505)

[Supplement 6: Symptoms used when assessing feeding intolerance 20](#_Toc193971506)

[Supplement 7: Gastric residual volume 20](#_Toc193971507)

[Supplement 8: Data on Guidelines 21](#_Toc193971508)

[Supplement 9: Preferred first choice of prokinetic agent by country 21](#_Toc193971509)

[Supplement 10: Data on other agents chosen as first or second choice 23](#_Toc193971510)

[Supplement 11: Duration of treatment for metoclopramide and erythromycin 23](#_Toc193971511)

[Supplement 12: Data on dose and frequency for domperidone and prucalopride 24](#_Toc193971512)

[Supplement 13: Data on preferred agent and symptoms in a future trial 26](#_Toc193971513)

# Supplement 1: Checklist for Reporting Of Survey Studies (CROSS)

| **Section/topic** | **Item** | **Item description** | **Reported on page #** |
| --- | --- | --- | --- |
| **Title and abstract** | | |  |
| Title and abstract | 1a | State the word “survey” along with a commonly used term in title or abstract to introduce the study’s design. | 1 |
|  | 1b | Provide an informative summary in the abstract, covering background, objectives, methods, findings/results, interpretation/discussion, and conclusions. | 3 |
| **Introduction** | | |  |
| Background | 2 | Provide a background about the rationale of study, what has been previously done, and why this survey is needed. | 4 |
| Purpose/aim | 3 | Identify specific purposes, aims, goals, or objectives of the study. | 4 |
| **Methods** | | |  |
| Study design | 4 | Specify the study design in the methods section with a commonly used term (e.g., cross-sectional or longitudinal). | 5 |
|  | 5a | Describe the questionnaire (e.g., number of sections, number of questions, number and names of instruments used). | 5 |
| Data collection methods | 5b | Describe all questionnaire instruments that were used in the survey to measure particular concepts. Report target population, reported validity and reliability information, scoring/classification procedure, and reference links (if any). | 5 |
|  | 5c | Provide information on pretesting of the questionnaire, if performed (in the article or in an online supplement). Report the method of pretesting, number of times questionnaire was pre-tested, number and demographics of participants used for pretesting, and the level of similarity of demographics between pre-testing participants and sample population. | 5 |
|  | 5d | Questionnaire if possible, should be fully provided (in the article, or as appendices or as an online supplement). | Supplement Appendix 2 |
| Sample characteristics | 6a | Describe the study population (i.e., background, locations, eligibility criteria for participant inclusion in survey, exclusion criteria). | 5,6 |
|  | 6b | Describe the sampling techniques used (e.g., single stage or multistage sampling, simple random sampling, stratified sampling, cluster sampling, convenience sampling). Specify the locations of sample participants whenever clustered sampling was applied. | 5,6 |
|  | 6c | Provide information on sample size, along with details of sample size calculation. | 7 |
|  | 6d | Describe how representative the sample is of the study population (or target -population if possible), particularly for population-based surveys. |  |
| Survey  administration | 7a | Provide information on modes of questionnaire administration, including the type and number of contacts, the location where the survey was conducted (e.g., outpatient room or by use of online tools, such as SurveyMonkey). | 5,6 and Supplementary Appendix 3 |
|  | 7b | Provide information of survey’s time frame, such as periods of recruitment, exposure, and follow-up days. | 5 |
|  | 7c | Provide information on the entry process:  –>For non-web-based surveys, provide approaches to minimize human error in data entry.  –>For web-based surveys, provide approaches to prevent “multiple participation” of participants. | 5 |
| Study preparation | 8 | Describe any preparation process before conducting the survey (e.g., interviewers’ training process, advertising the survey). | - |
| Ethical considerations | 9a | Provide information on ethical approval for the survey if obtained, including informed consent, institutional review board [IRB] approval, Helsinki declaration, and good clinical practice [GCP] declaration (as appropriate). | 5 |
|  | 9b | Provide information about survey anonymity and confidentiality and describe what mechanisms were used to protect unauthorized access. | 5 |
| Statistical  analysis | 10a | Describe statistical methods and analytical approach. Report the statistical software that was used for data analysis. | 6 |
|  | 10b | Report any modification of variables used in the analysis, along with reference (if available). | - |
|  | 10c | Report details about how missing data was handled. Include rate of missing items, missing data mechanism (i.e., missing completely at random [MCAR], missing at random [MAR] or missing not at random [MNAR]) and methods used to deal with missing data (e.g., multiple imputation). | 6 |
|  | 10d | State how non-response error was addressed. | 6 |
|  | 10e | For longitudinal surveys, state how loss to follow-up was addressed. | - |
|  | 10f | Indicate whether any methods such as weighting of items or propensity scores have been used to adjust for non-representativeness of the sample. | - |
|  | 10g | Describe any sensitivity analysis conducted. | - |
| **Results** | | |  |
| Respondent characteristics | 11a | Report numbers of individuals at each stage of the study. Consider using a flow diagram, if possible. | 7-9 |
|  | 11b | Provide reasons for non-participation at each stage, if possible. | - |
|  | 11c | Report response rate, present the definition of response rate or the formula used to calculate response rate. | Supplementary Appendix 4 |
|  | 11d | Provide information to define how unique visitors are determined. Report number of unique visitors along with relevant proportions (e.g., view proportion, participation proportion, completion proportion). | - |
| Descriptive  results | 12 | Provide characteristics of study participants, as well as information on potential confounders and assessed outcomes. | Table 1 |
| Main findings | 13a | Give unadjusted estimates and, if applicable, confounder-adjusted estimates along with 95% confidence intervals and p-values. | 7-9 |
|  | 13b | For multivariable analysis, provide information on the model building process, model fit statistics, and model assumptions (as appropriate). | - |
|  | 13c | Provide details about any sensitivity analysis performed. If there are considerable amount of missing data, report sensitivity analyses comparing the results of complete cases with that of the imputed dataset (if possible). | - |
| **Discussion** | | |  |
| Limitations | 14 | Discuss the limitations of the study, considering sources of potential biases and imprecisions, such as non-representativeness of sample, study design, important uncontrolled confounders. | 11 |
| Interpretations | 15 | Give a cautious overall interpretation of results, based on potential biases and imprecisions and suggest areas for future research. | 10-11 |
| Generalizability | 16 | Discuss the external validity of the results. | 11 |
| **Other sections** | | |  |
| Role of funding source | 17 | State whether any funding organization has had any roles in the survey’s design, implementation, and analysis. | 12 |
| Conflict of interest | 18 | Declare any potential conflict of interest. | 1 |
| Acknowledgements | 19 | Provide names of organizations/persons that are acknowledged along with their contribution to the research. | 12 |

#
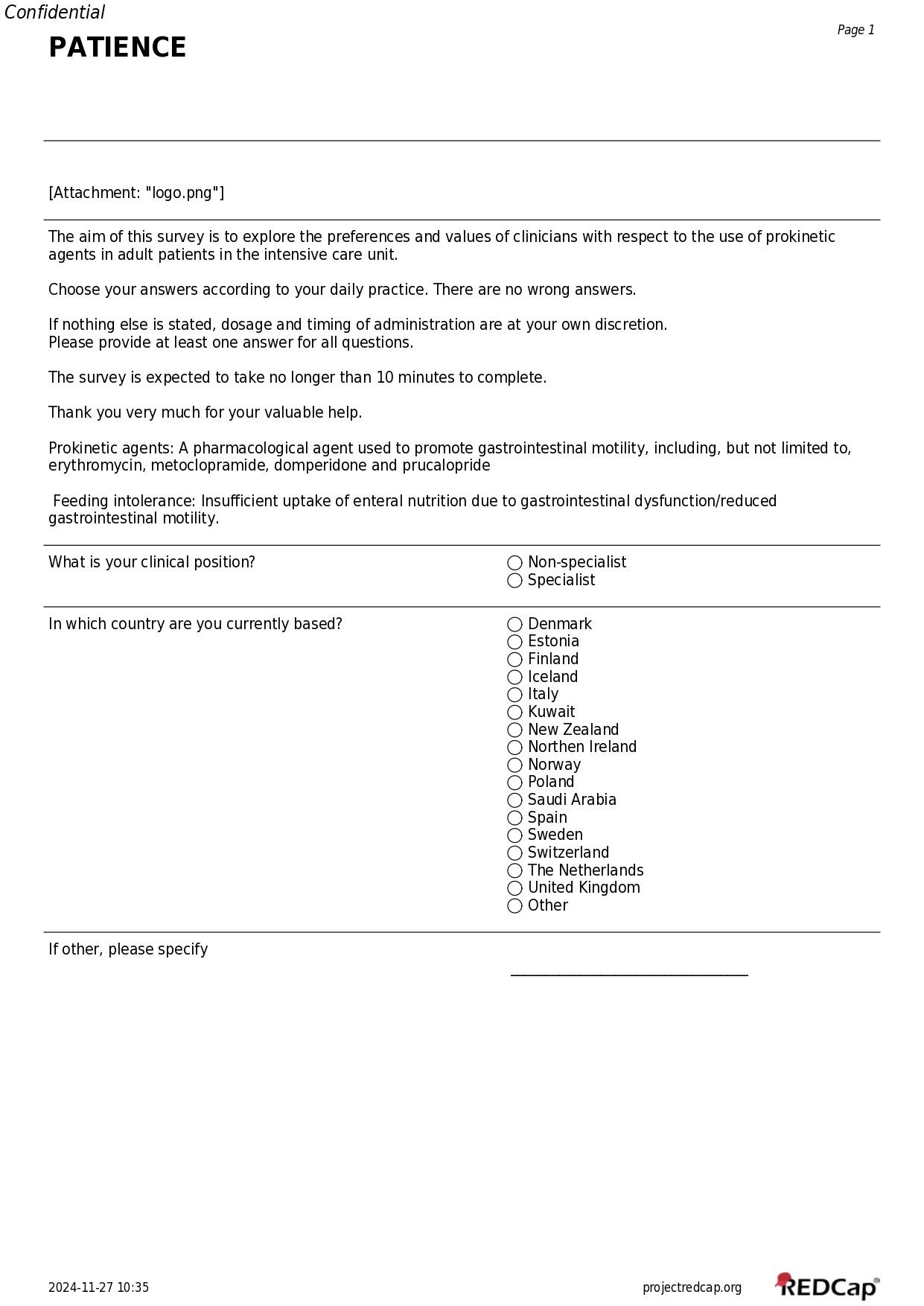
Supplement 2: The distributed survey


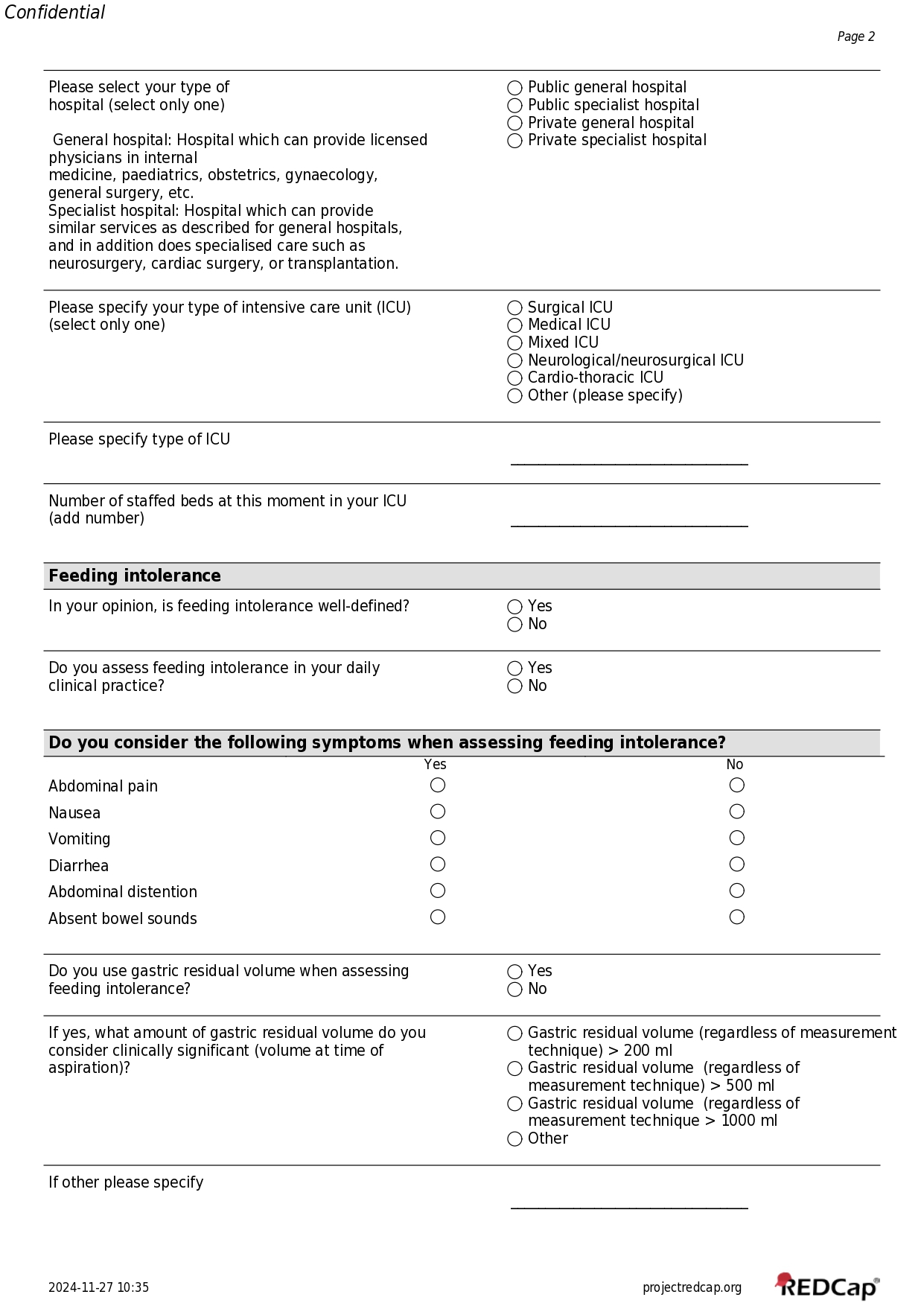

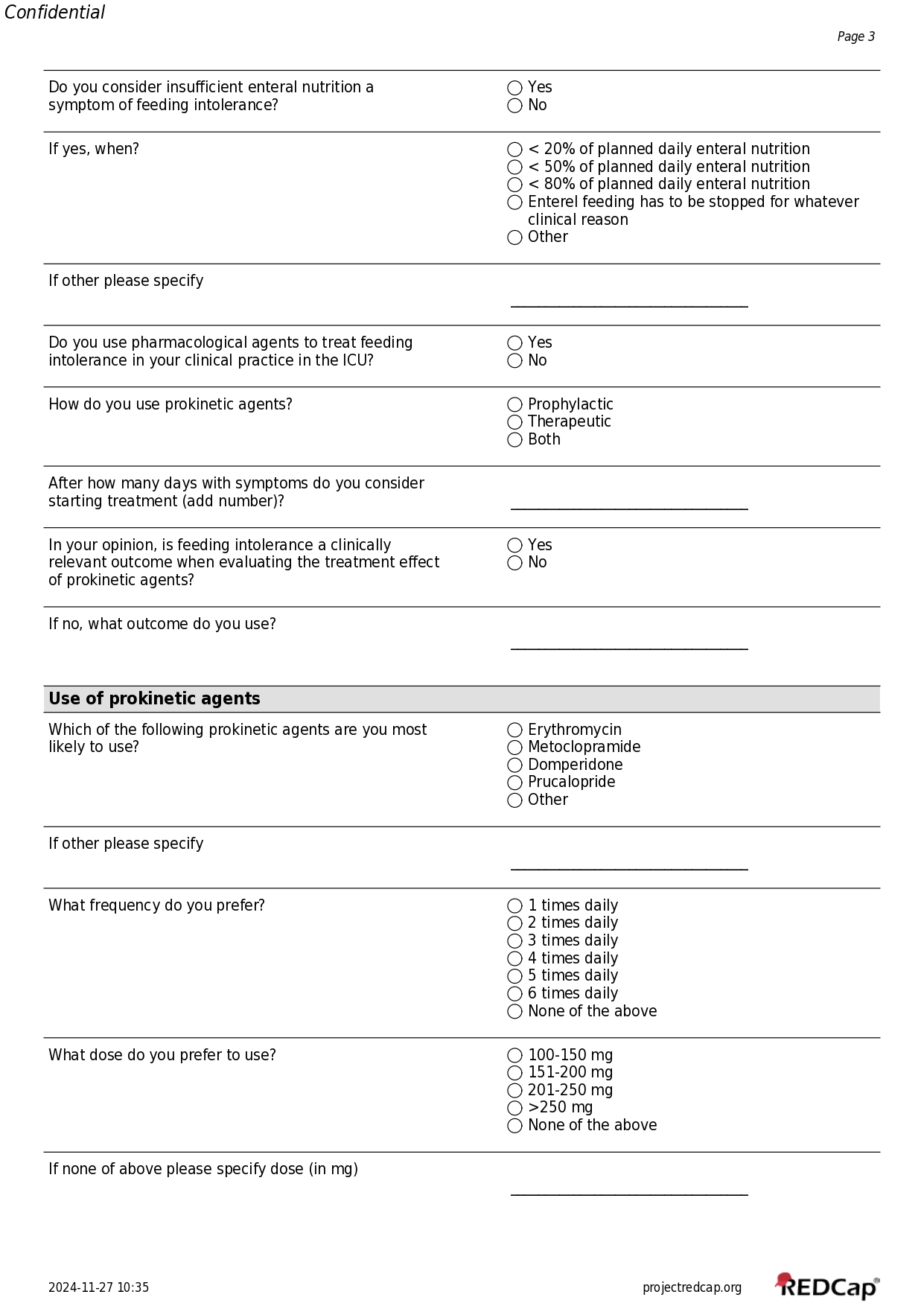

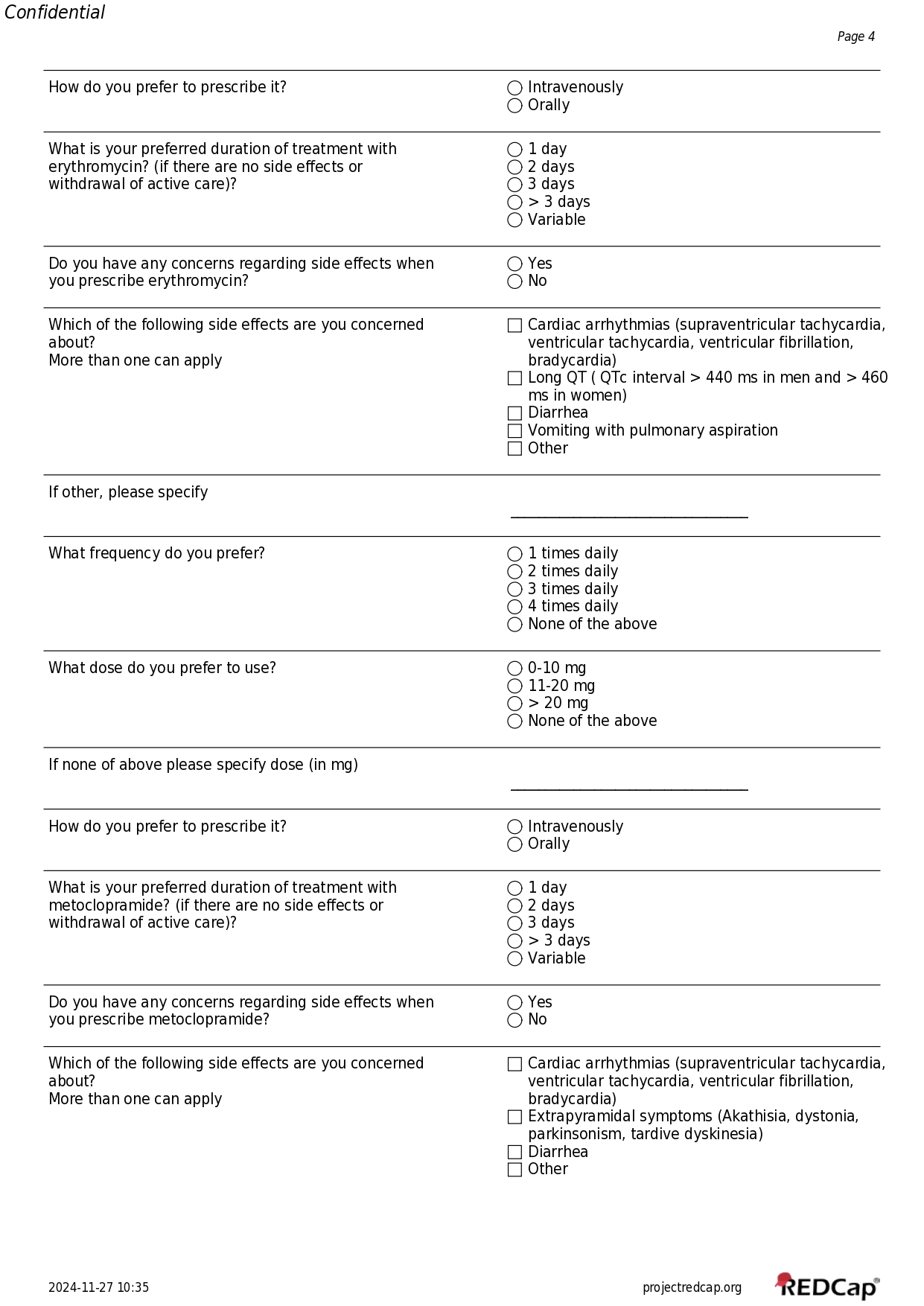

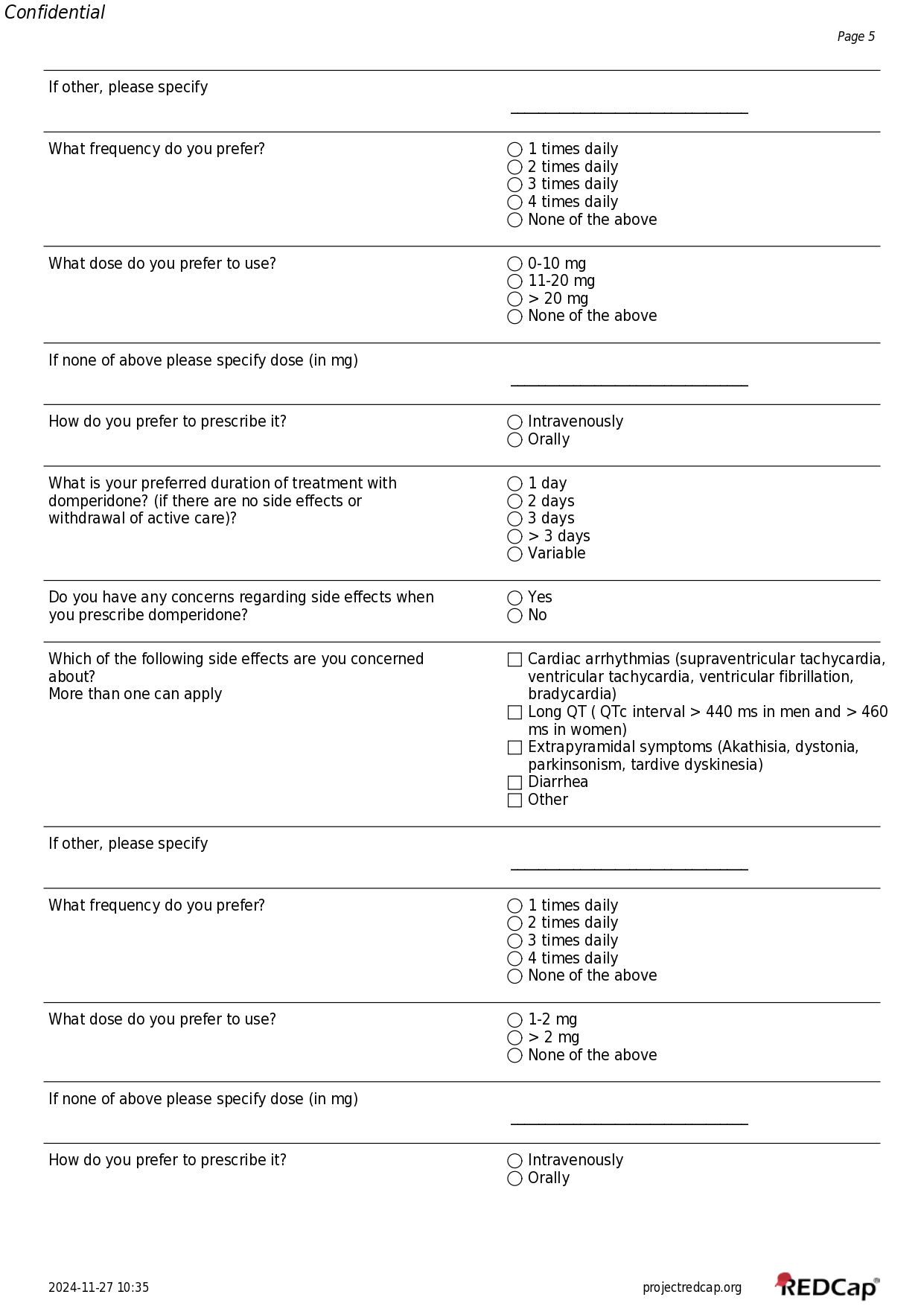

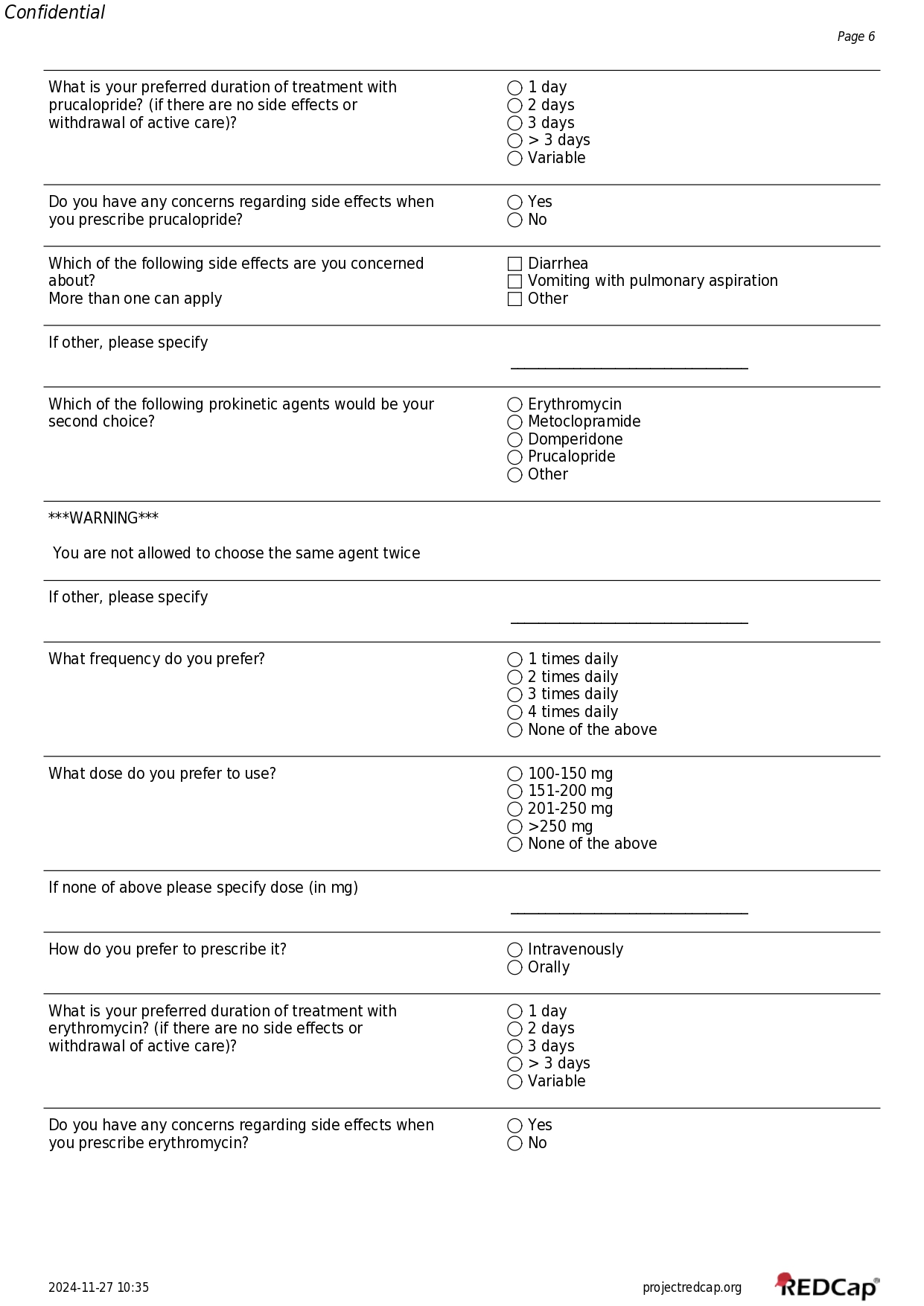

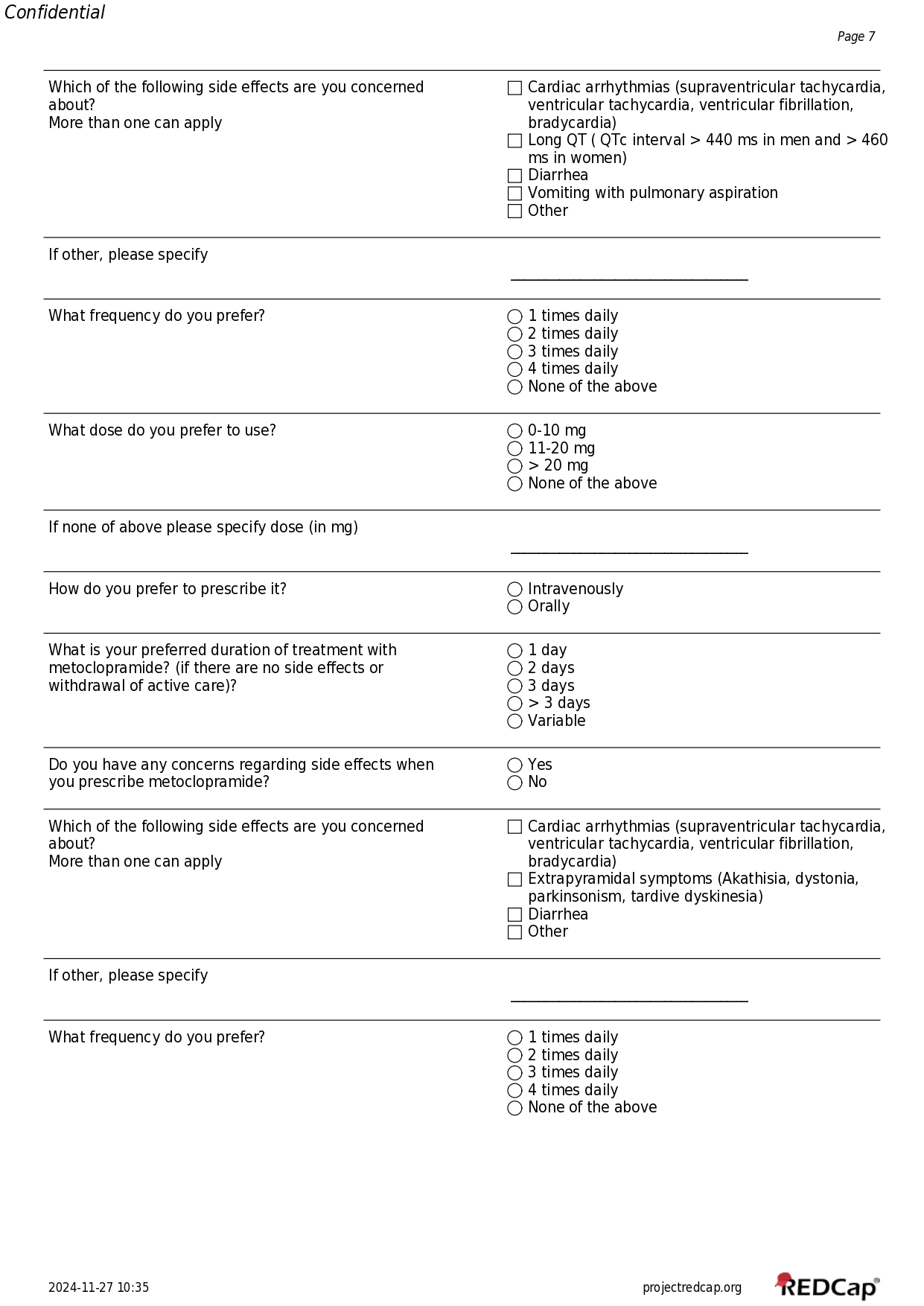

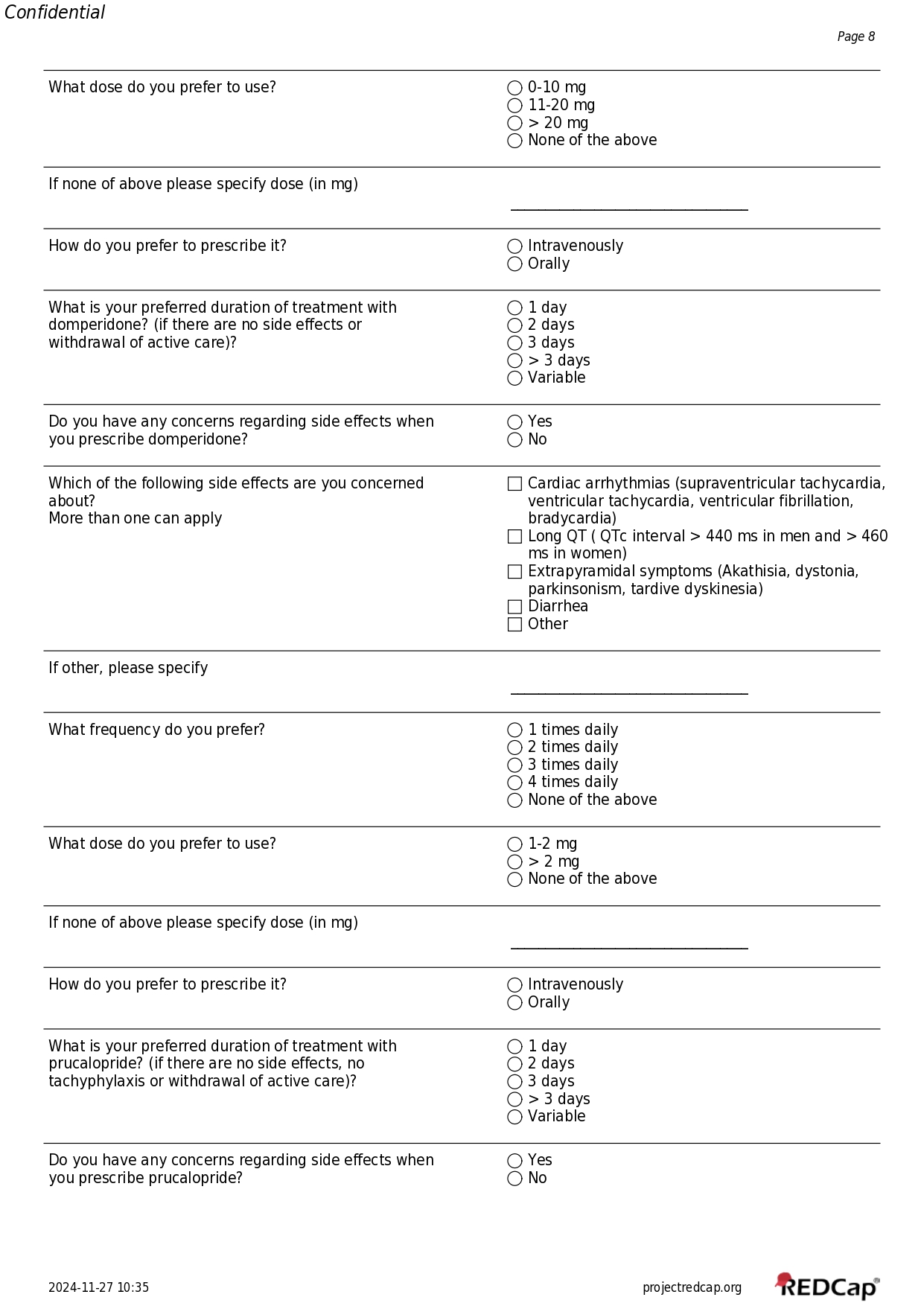

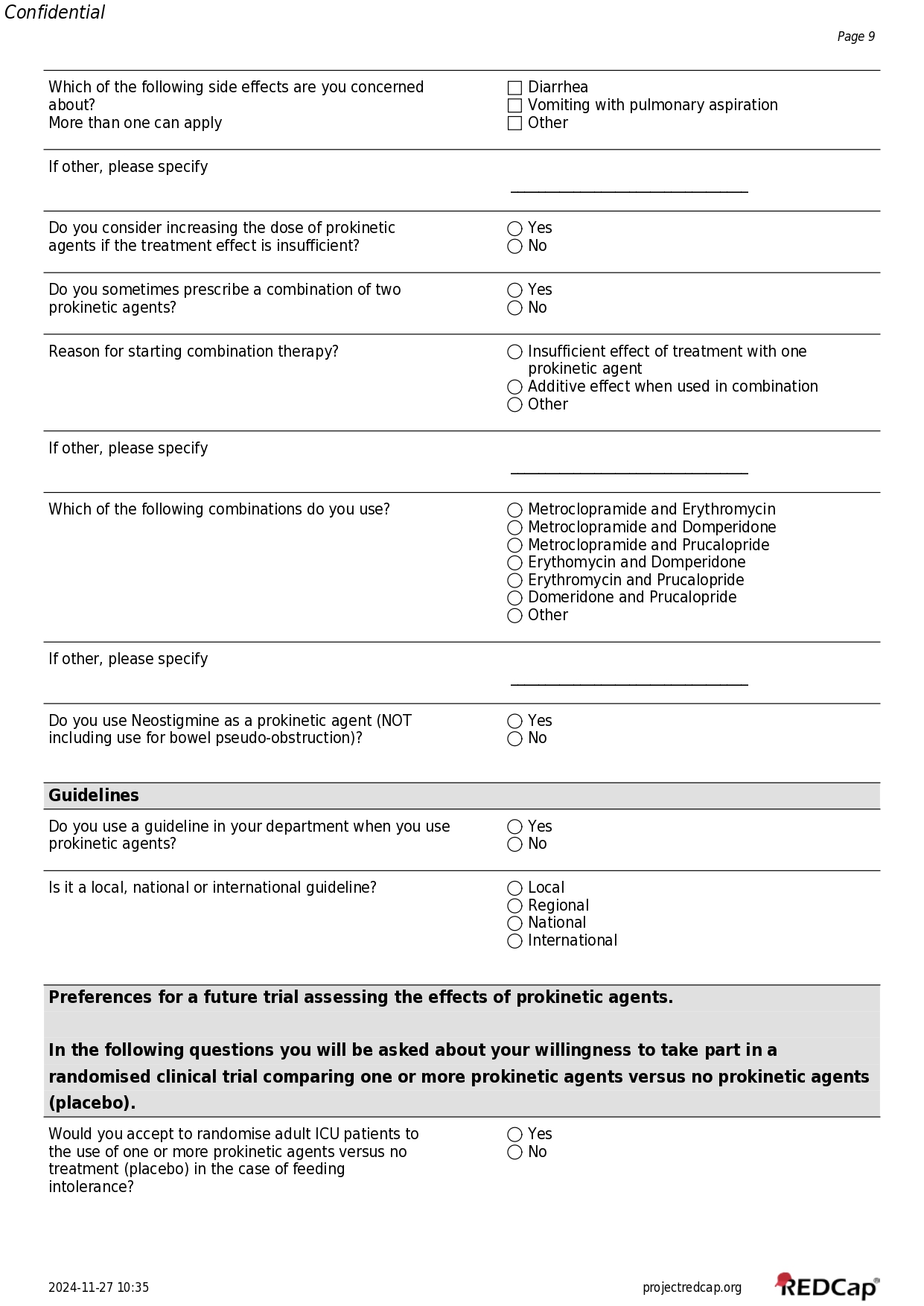

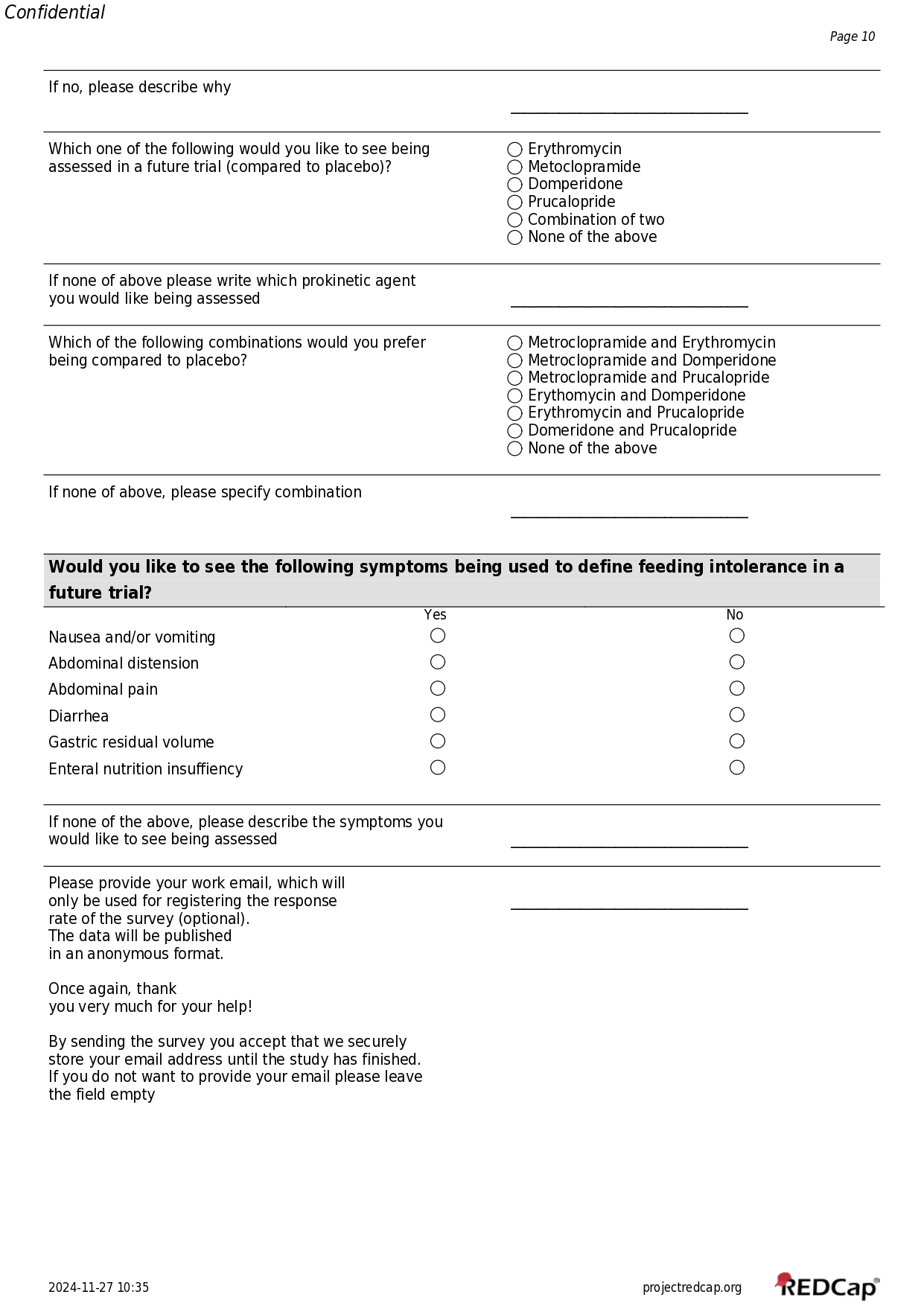


# Supplement 3: Participating countries, sites and investigators

| **Denmark** | |
| --- | --- |
| Department of Anaesthesiology and Intensive Care, Copenhagen University Hospital - Amager and Hvidovre, Hvidovre, Denmark | Christian Svendsen Juhl |
| Department of Anaesthesiology and Intensive Care, Copenhagen University Hospital - Bispebjerg and Frederiksberg Hospital, Copenhagen, Denmark | Theis Itenov |
| Department of Anaesthesiology and Intensive Care, Copenhagen University Hospital - Herlev-Gentofte Hospital, Gentofte, Denmark | Björn Anders Brand |
| Department of Anaesthesiology and Intensive Care, Copenhagen University Hospital - Herlev-Gentofte Hospital, Herlev, Denmark | Anne Sofie Andreasen |
| Department of Anaesthesiology and Intensive Care, Copenhagen University Hospital -North Zealand, Hilleroed, Denmark | Morten H. Bestle |
| Department of Anaesthesiology and Intensive Care, Holbaek Hospital, Holbaek, Denmark | Vera Crone |
| Department of Anaesthesiology and Intensive Care, Zealand University Hospital, Koege, Denmark | Tobias Browall Krogh |
| Department of Anaesthesiology and Intensive Care, Zealand University Hospital, Roskilde, Denmark | Andreas Bender Jonsson |
| Department of Anaesthesiology and Intensive Care, Aalborg University Hospital, Aalborg University, Aalborg, Denmark | Bodil Steen Rasmussen |
| Department of Anaesthesiology and Intensive Care, Aarhus University Hospital, Aarhus, Denmark | Steffen Christensen |
| Department of Cardiothoracic Anaesthesiology, Copenhagen University Hospital - Rigshospitalet, Copenhagen, Denmark | Peter Buhl |
| Department of Intensive Care, Copenhagen University Hospital - Rigshospitalet, Copenhagen, Denmark | Praleene Sivapalan |
| Department of Intensive Care, Gødstrup Hospital, Gødstrup, Denmark | Christopher Torp Lohse |
| Department of Anaesthesiology and Intensive Care, University Hospital of Southern Denmark, Kolding, Denmark | Anne Craveiro Brøchner |
| Department of Intensive Care, Odense University Hospital, Odense, Denmark | Jens Michelsen |
| Department of Intensive Care, Odense University Hospital, Svendborg, Denmark | Peter Martin Hansen |
| Department of Intensive Care, Randers Hospital, Randers, Denmark | Mathias Sinkbæk Thomsen |
| Department of Anaesthesiology and Intensive Care, Zealand University Hospital, Nykøbing Falster, Denmark | Kristian Reinhold Jauho |
| Department of Neuroanaesthesiology, Copenhagen University Hospital - Rigshospitalet, Copenhagen, Denmark | Sandra Lindkvist-Viggers |
| Department of Intensive Care, University Hospital of Southern Denmark, Aabenraa, Denmark | Thomas Strøm |
| Department of Anaesthesiology and Intensive Care, Lillebaelt Hospital, Vejle, Denmark | Jonas Elmer Pedersen |
| Department of Intensive Care, North Denmark Regional Hospital – Hjoerring, Denmark | Kjeld Damgaard |
| Department of Intensive Care, Viborg Regional Hospital, Viborg, Denmark | Therese Simonsen Straarup |

**International sites**

| **Department** | **Site investigators** |
| --- | --- |
| **Estonia** | |
| Department of Anaesthesiology and Intensive Care, Tartu University Hospital, Tartu, Estonia | Kadri Tamme |
| Centre of Anaesthesiology and Intensive Care, East Tallinn Central Hospital, Tallinn, Estonia | Maarja Hallik |
| Intensive Care Centre, North Estonia Medical Centre, Tallinn, Estonia | Liivi Maddison |
| **Finland** | |
| Tampere Heart Hospital, Tampere Finland | Timo Porkkala |
| Intensive Care Unit, Well-Being Services County of Kanta-Häme, Hämeenlinna, Finland | Ari Alaspää |
| Intensive Care Unit, Well-Being Services County of Satakunta, Pori, Finland | Björn Jäschke |
| Intensive Care Unit, Turku University Hospital, Wellbeing Services County of Southwest Finland, Turku, Finland | Mika Valtonen |
| Intensive Care Units, Helsinki University Hospital, Helsinki, Finland | Minna Bäcklund |
| Intensive Care Unit, Tampere University Hospital, Wellbeing Services County of Pirkanmaa, Tampere, Finland | Ville Jalkanen |
| Intensive Care Unit, Päijät-Häme Central Hospital, Lahti, Finland | Marika Lähde |
| Intensive Care Unit, North Carelia Central Hospital, Joensuu, Finland | Sampsa Suvela |
| Intensive Care Unit, Wellbeing Services County of Kainuu, Kajaani, Finland | Panu Piirainen |
| Intensive Care Unit, Kuopio University Hospital, Wellbeing Services County of North Savo, Kuopio, Finland | Stapani Bendel |
| Intensive Care Unit, Wellbeing Services County of Ostrobothnia, Vaasa, Finland | Simo-Pekka Koivisto |
| Intensive Care Unit, Wellbeing Services County of Lapland,Rovaniemi, Finland | Anna Eklund |
| Intensive Care Unit, Wellbeing Services County of Kymenlaakso, Kotka, Finland | Jussi Pentti |
| Intensive Care Unit, Wellbeing Services County of Central Uusimaa, Hyvinkää, Finland | Elina Riihioja |
| **Iceland** | |
| Department of Anaesthesiology and Intensive Care Medicine, Landspital - The National University Hospital of Iceland, Reykjavik, Iceland | Martin I. Sigurdsson |
| **Italy** | |
| Department of Anaesthesiology and Intensive Care, IRCCS Istituto Clinico Humanitas, Humanitas University, Milan, Italy | Maurizio Cecconi |
| **Kuwait** | |
| Department of Anesthesia, Critical Care Medicine and Pain Medicine, Al-Amiri Hospital, Kuwait City, Kuwait. | Abdulrahman Al-Fares |
| **New Zealand** | |
| Intensive Care Unit, Wellington Hospital, Wellington, New Zealand | Paul J Young |
| **Norway** | |
| Department of Intensive Care, Oslo Universitetssykehus (Rikshospitalet) | Lasse Grønningsæter |
| Department of Intensive Care, Helse Møre og Romsdal (Ålesund) | Finn Husøy Andersen |
| Department of Intensive Care, Akershus Universitetssykehus | Ole Kristian Fossum |
| Department of Intensive Care, Sykehuset i Vestfold | Thor Andreas Moe Slinning |
| Department of Intensive Care, Sykehuset Østfold HF | Peter Holm |
| Department of Intensive Care, Sørlandet sykehus HF (Arendal) | Vegard Dokka |
| Department of Intensive Care, Helse Stavanger HF | Kristian Strand |
| Department of Intensive Care, Diakonhjemmet sykehus | Aasmund Godø |
| Department of Intensive Care, Lovisenberg Diakonale sykehus | Tron Blien |
| **Poland** |  |
| Center for Intensive Care and Perioperative Medicine, Jagiellonian University Medical College, Krakow, Poland | Wojciech Szczeklik |
| **Saudi Arabia** | |
| Department of Critical Care Medicine, King Saud University, Riyadh, Saudi Arabia | Waleed Alhazzani |
| **Spain** | |
| Department of Intensive Care,Vall d'Hebron Hospital Universitari, Vall d'Hebron Barcelona, Hospital Campus, Barcelona, Spain | Ricard Ferrer |
| **Sweden** | |
| Department of Intensive Care, Danderyd Hospital, Stockholm, Sweden | Olof Wall |
| **Switzerland** | |
| Department of Intensive Care, Inselspital, Bern University Hospital and University of Bern, Bern, Switzerland | Carmen Pfortmueller |
| Department of Intensive Care, Cantonal Hospital of St.Gallen | Urs Pietsch |
| Department of Intensive Care, University Hospital of Basel | Alexa Holliger |
| **The Netherlands** | |
| Dept. of Critical Care, University Medical Center Groningen, University of Groningen, Groningen, The Netherlands | Eric Keus |
| **The United Kingdom** | |
| King's College London, Guy's & St Thomas' Hospital London, England | Marlies Ostermann |
| **Northern Ireland** |  |
| Department of Critical Care, Belfast Health and Social Care Trust, Northern Ireland | Chris McGrath |

# Supplement 4: Data on response rate

**Table 1: Response rate per country**

| **Country** | **Response rate** |
| --- | --- |
| Denmark | 289/722 (40%) |
| Great Britain | 38/88 (43%) |
| Estonia | 35/95 (37%) |
| Finland | 55/246 (22%) |
| The Netherlands | 9/9 (100%) |
| Iceland | 12/37 (32%) |
| Italy | 16/200 (8%) |
| Kuwait | 17/60 (28%) |
| New Zealand | 11/41 (27%) |
| Norway | 61/336 (18%) |
| Northern Ireland | 26/87 (30%) |
| Poland | 37/55 (67%) |
| Spain | 76/3365 (2%) * |
| Saudi Arabia | 53/219 (24%) |
| Sweden | 57/230 (25%) |
| Switzerland | 33/140 (24 %) |

***Table 1*** ******The survey was distributed to a large network of people shortly before it closed. Therefore, Spain's response rate was not included in the calculation of the overall response rate.***

# Supplement 5: Missingness in the main questions

*Missingness in the 13 main survey questions (the questions that everyone was expected to answer regardless of their previous responses)*

| **Variable** | **Missing responses**  **n=830 (%)** |
| --- | --- |
| **Question 1**  What is your clinical position | 2 (0.2%) |
| **Question 2**  In which country are you currently based? | 2 (0.2%) |
| **Question 3**  Type of hospital | 0 (0.0%) |
| **Question 4**  Type of ICU | 1 (0.1%) |
| **Question 5**  Number of staffed bed | 14 (1.6%) |
| **Question 6**  In your opinion, is feeding intolerance well-defined? | 2 (0.2%) |
| **Question 7**  Do you assess feeding intolerance in your daily clinical practice? | 3 (0.4% |
| **Question 8**  Do you use pharmacological agents to treat feeding intolerance in your clinical practice in the ICU? | 2 (0.2%) |
| **Question 9**  In your opinion, is feeding intolerance a clinically relevant outcome when evaluating the treatment effect of prokinetic agents? | 3 (0.4%) |
| **Question 10**  Do you consider increasing dose of prokinetic agents if the treatment effect is insufficient? | 4 (0.5%) |
| **Question 11**  Do you use Neostigmin as a prokinetic agent (NOT including use for bowel pseudoobstruction)? | 5 (0.6%) |
| **Question 12**  Do you use a guideline in your department when you use prokinetic agents? | 6 (0.7%) |
| **Question 13**  Would you accept to randomise adult ICU patients to the use of one or more prokinetic agents versus no treatment (placebo) in the case of feeding intolerance? | 5 (0.6%) |

*Missingness for the main questions posed to the respondents answering “yes” to assessing feeding intolerance in their daily clinical practice*

| **Variable** | **Missing responses**  **n=747 (%)** |
| --- | --- |
| **Question 1**  Do you consider the following symptoms when assessing feeding intolerance? | Abdominal pain: 13 (1.7%)  Nausea: 10 (1.3%)  Vomiting: 5 (0.7%)  Diarrhea:12 (1.6%)  Abdominal distention: 8 (1.0%)  Absent bowel sounds: 14 (1.8%) |
| **Question 2**  Do you use gastric residual volume when assessing feeding intolerance? | 6 (0.8%) |
| **Question 3**  Do you consider insufficient enteral nutrition a symptom of feeding intolerance? | 7 (0.9%) |

*Missingness for the main questions posed to the respondents answering ” yes” to using pharmacological agents to treat feeding intolerance in their clinical practice*

| **Variable** | **Missing responses**  **n=773 (%)** |
| --- | --- |
| **Question 1**  How do you use prokinetic  Agents? | 3 (0.3%) |
| **Question 2**  After how many days do you consider starting treatment? | 11 (1.4%) |
| **Question 3**  Which of the following prokinetic agents are you most likely to use? | 4 (0.5%) |
| **Question 4**  Which of the following prokinetic agents would be your second choice? | 6 (0.8%) |
| **Question 5**  Do you sometimes prescribe a combination of two prokinetic agents | 6 (0.8%) |

*Missingness for the main questions posed to the respondents answering” yes” to accepting to randomise ICU patients in a clinical trial*

| **Variable** | **Missingness**  **n=751 (%)** |
| --- | --- |
| **Question 1**  Which of the following would you like to see being assessed in a future trial? | 7 (0.9%) |

# Supplement 6: Symptoms used when assessing feeding intolerance

**Table 2: Symptoms considered when assessing feeding intolerance**

| **Symptoms** | **Percentages (yes respondents/total respondents)** |
| --- | --- |
| Vomiting | 98.5% (731/742) |
| Abdominal distention | 90.1% (666/739) |
| Nausea | 84.1% (620/737) |
| Diarrhea | 77.3% (568/735) |
| Abdominal pain | 70.6% (518/734) |
| Absent bowel sounds | 69.0%% (506/733) |

**Table 2. *Respondents could select more than one symptom. Each symptom was presented as a yes/no question, and some respondents only provided answers for specific symptoms*.**

# Supplement 7: Gastric residual volume

**Figure 1: Amount of gastric residual volume considered clinically significant.**

**Fig 1. *The category ‘Other’ ranged from 250 ml to 400 ml***

# Supplement 8: Data on Guidelines

**Figure 2: Type of guideline**


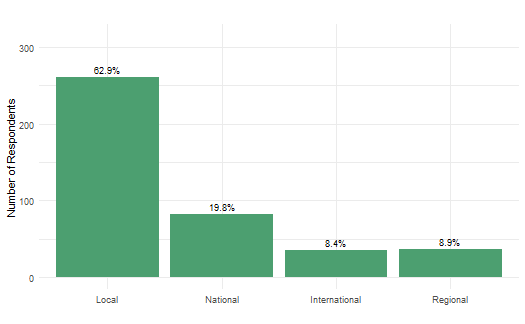


**Fig 2:** ***Total number of respondents N=415***

# Supplement 9: Preferred first choice of prokinetic agent by country

**
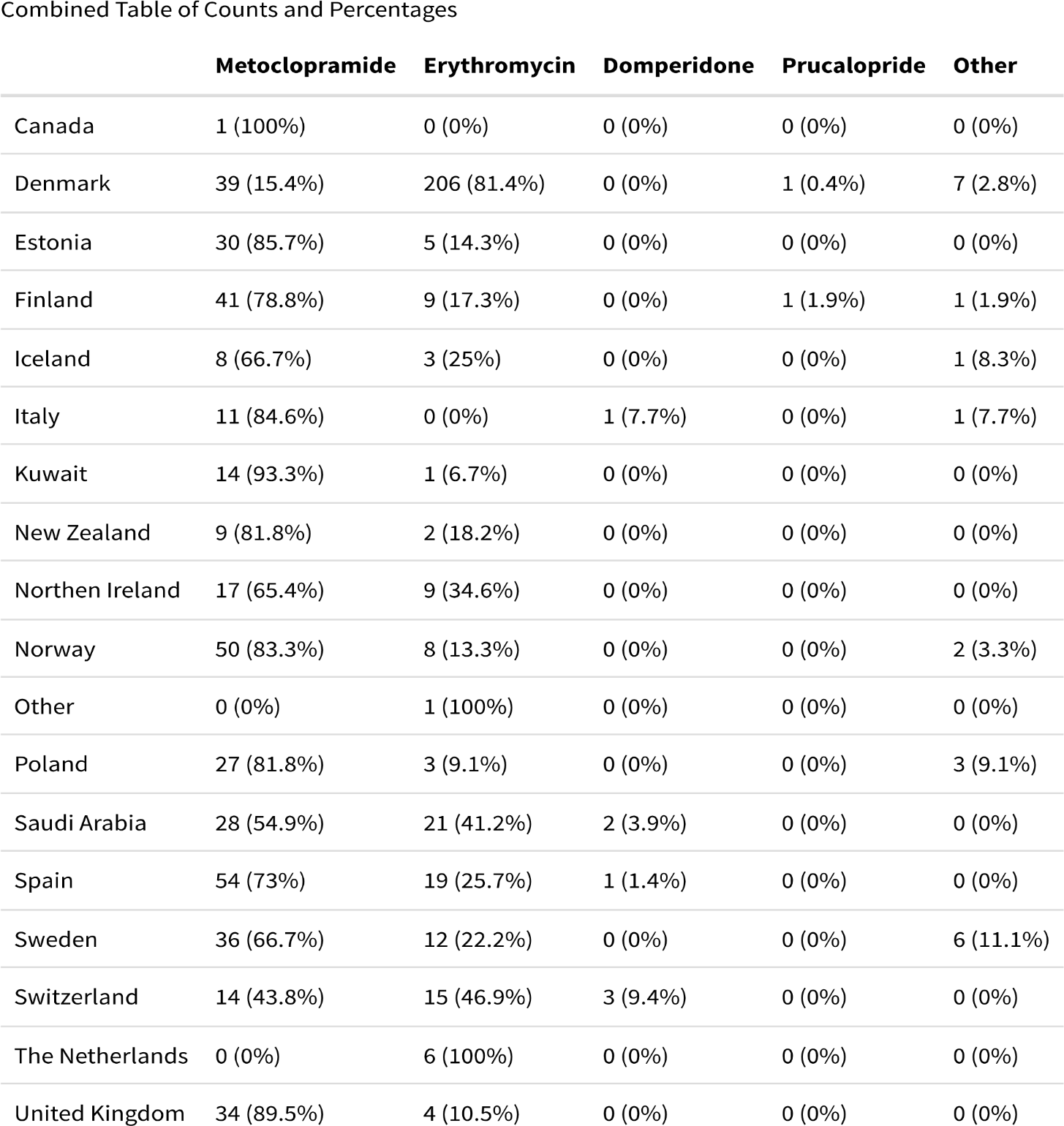
Table 3: Preferred first choice of prokinetic agent by country**

# Supplement 10: Data on other agents chosen as first or second choice

**Table 4**

| **Agent** | **N = 54 (%)** |
| --- | --- |
| Laxatives (macrogol, actilax,  Magnesium hydroxide, Sodium picosulfate, bisacodyl) | 22 (40.7%) |
| Neostigmine | 12 (22.2%) |
| Itopride | 9 (16.7%) |
| Methylnaltrexone bromide (Relistor) | 4 (7.4%) |
| Naloxone | 4 (7.4%) |
| Ondansetron | 2 (3.7%) |
| Pyridostigmine | 1 (1.9%) |

**Table 4:** ***Other agents mentioned as first or second choice. Some respondents provided answers for both first and second choices, resulting in them being counted twice in the table****.*

# Supplement 11: Duration of treatment for metoclopramide and erythromycin

**Figure 3: Preferred duration of treatment for metoclopramide**

| **Duration of treatment** | **1 day** | **2 days** | **3 days** | **> 3 days** | **Variable** |
| --- | --- | --- | --- | --- | --- |
| Percentages (%) | 1.8 % | 7.4 % | 45.3 % | 14.0 % | 31.4 % |

**Figure 4: Preferred duration of treatment for erythromycin**

| **Duration of treatment** | **1 day** | **2 days** | **3 days** | **> 3 days** | **Variable** |
| --- | --- | --- | --- | --- | --- |
| Percentages (%) | 2.9% | 8.0% | 55.6% | 8.5% | 24.8% |

# Supplement 12: Data on dose and frequency for domperidone and prucalopride

**Figure 5a: The reported doses and frequencies of domperidone, selected as the preferred first or second choice.**


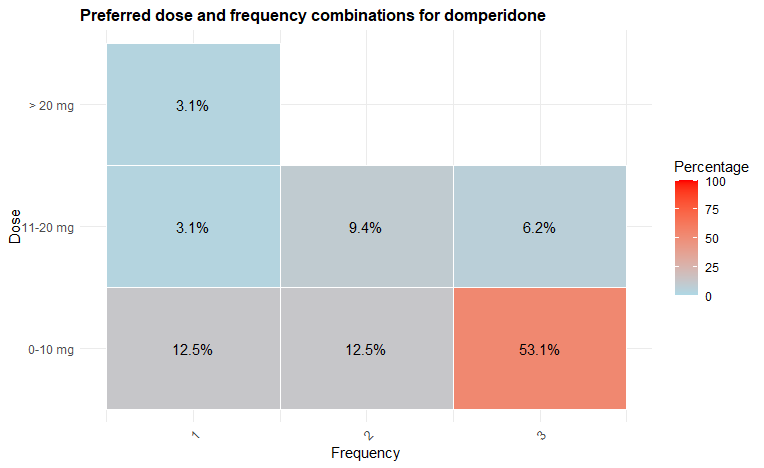


| **Duration of treatment** | **1 day** | **2 days** | **3 days** | **> 3 days** | **Variable** |
| --- | --- | --- | --- | --- | --- |
| Percentages (%) | 3.0 % | 12.0 % | 33.3 % | 27.2 % | 24.2% |

**Figure 5a*. Total number of respondents = 33***

**Figure 5b: Preferred duration of treatment for domperidone**


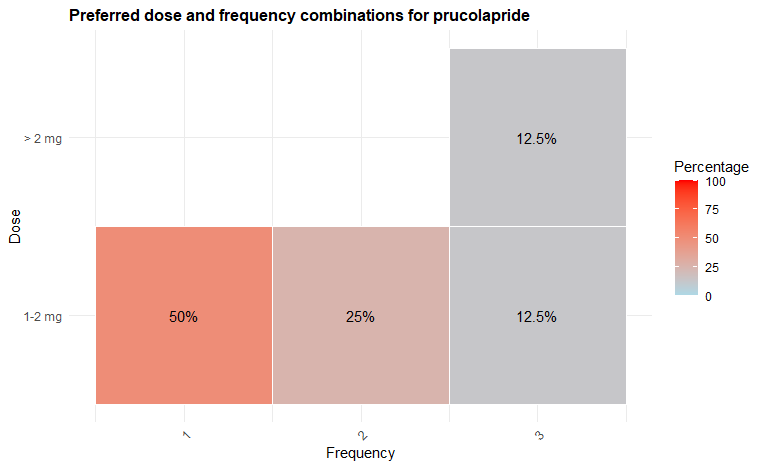
**Figure 6a: The reported doses and frequencies of prucolapride selected as preferred first or second choice*.***

**Figure 6a*. Total number of respondents = 8***

| **Duration of treatment** | **1 day** | **2 days** | **3 days** | **> 3 days** | **Variable** |
| --- | --- | --- | --- | --- | --- |
| Percentages (%) | 0.0 % | 11.1 % | 11.1 % | 11.1 % | 54.5 % |

**Figure 6b: Preferred duration of treatment for prucolapride**

# Supplement 13: Data on preferred agent and symptoms in a future trial

***
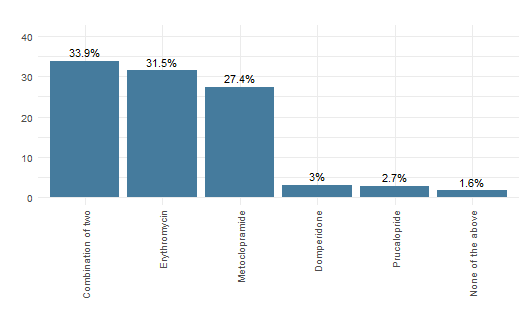
***

**Fig 7*. Number of respondents = 744. For those who selected “none of the above,” one suggested macrogol, one itopride, and one methylnaltrexone, and the remaining reported “no opinion” or left the field blank.***

**Table 5: Preferred symptoms to be used in the definition of feeding intolerance in a future trial**

| **Symptoms** | **Percentages (yes respondents/total respondents)** |
| --- | --- |
| Gastric residual volume | 93.9% (769/819) |
| Enteral nutrition insufficiency | 85.4% (691/809) |
| Nausea and/or vomiting | 88.6% (723/816) |
| Abdominal distention | 76.7% (623/812) |
| Abdominal pain | 64.9% (518/798) |
| Diarrhea | 64.7% (515/796) |

**Table 5. *Respondents could select more than one symptom. Each symptom was presented as a yes/no question, and some respondents only provided answers for specific symptoms*.**
